# Transformer Decoder Learns from a Pretrained Protein Language Model to Generate Ligands with High Affinity

**DOI:** 10.1021/acs.jcim.4c02019

**Published:** 2025-01-28

**Authors:** Teresa Maria Creanza, Domenico Alberga, Cosimo Patruno, Giuseppe Felice Mangiatordi, Nicola Ancona

**Affiliations:** † Institute of Intelligent Industrial Technologies and Systems for Advanced Manufacturing, 9327 Consiglio Nazionale delle Ricerche, Via G. Amendola, 122/d, Bari 70126, Italy; ‡ Institute of Crystallography, Consiglio Nazionale delle Ricerche, Via G. Amendola, 122/d, Bari 70126, Italy

## Abstract

The drug discovery process can be significantly accelerated by using deep learning methods to suggest molecules with druglike features and, more importantly, that are good candidates to bind specific proteins of interest. We present a novel deep learning generative model, Prot2Drug, that learns to generate ligands binding specific targets leveraging (i) the information carried by a pretrained protein language model and (ii) the ability of transformers to capitalize the knowledge gathered from thousands of protein–ligand interactions. The embedding unveils the receipt to follow for designing molecules binding a given protein, and Prot2Drug translates such instructions by using the syntax of the molecular language generating novel compounds which are predicted to have favorable physicochemical properties and high affinity toward specific targets. Moreover, Prot2Drug reproduced numerous known interactions between compounds and proteins used for generating them and suggested novel protein targets for known compounds, indicating potential drug repurposing strategies. Remarkably, Prot2Drug facilitates the design of promising ligands even for protein targets with limited or no information about their ligands or 3D structure.

## Graphical abstract

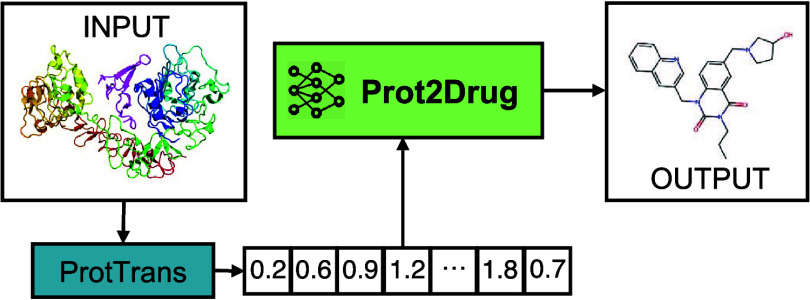

## Introduction

A challenging task in cheminformatics is finding bioactive compounds with given chemical properties that can bind a specific protein. Virtual screening^1^ and de novo design^2^ are two common techniques suited to face this problem. Virtual screening encompasses in silico approaches used in drug discovery to search libraries of billions of small molecules to identify those structures that are most likely to bind a drug target. De novo design aims to explore unknown regions of chemical space with the primary objective of producing ligands with specific chemical properties that bind a given protein target. Recently, deep learning models have made significant advances in de novo drug design by transferring relevant results and ideas from the field of natural language processing^3^ to cheminformatics. The main idea is to develop generative models that learn a data probability distribution by training on a large corpus of observations, enabling them to generate new data belonging to the same distribution.^4,5^ Many generative models developed in the field of de novo drug design use simplified molecular input line entry system (SMILES) as molecular representation and adopt Recurrent Neural Network (RNN)^6^ with LSTM units^7^ as a deep learning architecture for its ability to manage variable length input. Gupta et al.^8^ developed a generative RNN model containing LSTM units to learn the SMILES language syntax and generate valid small molecules. Moreover, they fine-tuned the model by further training on smaller subsets of selected compounds, with the objective of adapting the model to produce compounds binding to a specific target. Although their approach produced valid SMILES, its applicability is limited by the number of known ligands of the selected protein and cannot be applied to protein targets with few known ligands. Olivecrona et al.^9^ proposed a similar approach and adopted Reinforcement Learning^10^ as fine-tuning method successfully generating molecules active against the dopamine receptor type 2. Creanza et al. designed a data-driven deep learning model able to generate similar bioactive compounds starting from a single user-defined query molecule.^11^ Even though the model is suitable for performing de novo design even in low-data regimes, it generates druglike analogues of the query molecule, spanning only limited regions of the chemical space. Autoencoder models provide an alternative deep learning architecture widely used in de novo drug design.^12,13^

Although these architectures have enabled significant progress in the field of de novo design, they use the information on the target protein only implicitly through the knowledge of the binding molecules. Grechishnikova^14^ in her seminal paper indicated a new avenue of research and suggested a strategy for generating ligands explicitly leveraging the information embedded in the primary sequence of the target protein. To this end, she set the problem of de novo drug design as a machine translation problem between the amino acid language and the SMILES representation of the molecule. She trained a Transformer model^15^ on data sets of (*protein*, *small molecule*) interacting pairs for generating new ligands. Despite the indisputable novelty of the work and the elegance of the proposed strategy, the method suffered from two main aspects. The model complexity was extremely high because the model had to simultaneously manage the translation from a source language, including also amino acid sequences with more than 2000 characters in length, to a target language. Moreover, the number of generated SMILES strings was small, even expanding the beam search size to ten. In this framework, the recent paper by Chen et al.^16^ is worth mentioning. The authors proposed DeepTarget, an extremely complex deep learning model composed of (i) a module for generating embeddings from the input amino acid sequence of the target protein, (ii) a module for inferring potential features of the synthesized molecule, and (iii) a final module for generating binding molecules. Although the authors addressed the problem of increasing the number of generated molecules, their work focused only on two target proteins, and an analysis of results on a larger scale was missing.

In this paper, we present Prot2Drug, a novel and simple deep learning model for de novo drug design which generates novel potential ligands of a protein target by relying on its sequence embedding only. Our method successfully leverages recent advances of pretrained protein language models^17^ and the ability of transformer to capture long-range dependencies in sequences, providing state-of-the-art results in different domains including life science.^18^ The proposed approach is an example of implementation of a long-standing paradigm in machine learning in which informative features extracted from the input can enhance the power of simple models. Indeed, our model is composed of a simple two-layer transformer decoder and the ProtTrans distributed representation of the proteins^17^ is given directly as input to the decoder. In our context where the knowledge of drug–target interacting pairs is limited, using pretrained protein language models allows us to borrow strength from the much larger corpus of single protein sequences.^19^ In particular, as we show in the analysis of our results, pretraining is especially beneficial when the number of known ligands for a protein is small or when the query protein is unknown to Prot2Drug. Remarkably, for assessing the validity of our model we provide a large-scale analysis on 1734 human protein targets by using ConPLex,^20^ a recent open source, sequence-based drug–target interaction (DTI) prediction method which provides state-of-the-art performances. Moreover, as suggested,^21^ we determine the model’s ability to exactly reproduce known active compounds not encountered during training. Finally, we evaluate the design capacity of the model and its potential to generate candidate compounds with high diversity compared to those of the known compounds. The main contributions of our work are as follows:

- we present a generative method that designs from scratch specific novel ligands starting only from protein sequence embeddings. Prot2Drug capitalizes on the strengths of both ProtTrans distributed representation of the proteins and the knowledge of thousands of protein–ligand interactions from the ChEMBL database;^22^
- we provide a large-scale analysis of 1734 human protein targets and generate more than 4.5 million new compounds having promising physicochemical properties to be potential drugs;
- the new molecules *l*_*A*_ generated by Prot2Drug by using protein targets *A* are good candidates to bind *A* because exhibit predicted binding scores greater than random molecules and *comparable* to the ones assessed on known protein–ligand pairs;
- Prot2Drug reproduced 138 known compounds binding the same protein targets used for generating them, never seen during the training phase. For example, we considered the molecules generated from the protein representation of C-X-C chemokine receptor type 2 (CXCR2) that is a key regulator that drives immune suppression and can induce inflammation in tumor microenvironments and targeting CXCR2 has shown promising results in different solid tumors.^23^ Prot2Drug reproduced two molecules (CHEMBL563855 and CHEMBL564029) that are known ligands of CXCR2. Notice that CXCR2 and all its ligands were unknown to the model as unseen during learning. The ability of Prot2Drug to reproduce known ligands makes us confident that the novel generated molecules are good candidate ligands of the input proteins that merit further investigations.
- Prot2Drug reproduced 988 known bioactive compounds binding protein targets different from the ones used for generating them. Examples of compounds in this class are some known ligands of the Epidermal growth factor receptor (EGFR). One of these molecules (CHEMBL4208811) was generated by Prot2Drug from Receptor tyrosine-protein kinase erbB-2 (ERBB2) protein suggesting investigating the interaction between ERBB2 and this ligand. EGFR and ERBB2 have been shown to be involved in many aspects of oncogenic progression and are attractive targets for cancer therapies.^24^ Moreover, for some input proteins, Prot2Drug generated molecules that are FDA-approved small molecules and known binders of different proteins. Since our data suggest these input proteins as candidate targets of the known drugs, they may help identify off-targets which may result in adverse drug reactions and safety issues or suggest new candidates for repurposing the drugs.
- Finally, we assessed the ability of Prot2Drug to generate promising candidates for specific targets by using an independent structure-based approach (molecular docking) on two proteins involved in cancer and neurodegenerative diseases.

## Methods

### Data

The original data set consisted of 338,760 pairs, each comprising a protein primary sequence and the SMILES code of a ligand with known biological activity toward that protein. The data were retrieved by querying the entire ChEMBL33 database^22^ and retaining only those entries that met the following criteria: (i) “organism” = “*Homo sapiens*”; (ii) “assay_type” = “B”; (iii) “*K_i_*” or “IC50” value less than or equal to 100 nM; (iv) no “data_validity_comment”; and (v) “molecule_type” = “Small molecule”. The selected pairs underwent further preparation through a data curation pipeline applied to the SMILES sequences, as described in our previous works:^11,25,26^ (i) removal of stereoisomerism; (ii) desalting and neutralizing all entries; (iii) elimination of inorganic and metal atom compounds; (iv) removal of compounds containing elements other than H, C, N, O, F, Br, I, Cl, P, and S; (v) conversion of all entries into neutralized canonical SMILES; and (vi) exclusion of SMILES with character counts in the bottom 2.5% or top 2.5%. It is worth noting that the selected threshold (much more stringent than commonly used thresholds, such as 1 μM) addresses the need to minimize background noise in the data and to develop a model capable of generating molecules with significant therapeutic interest. The data set comprised 1753 unique protein sequences and 223,764 unique ligands. The protein sequences were represented by using ProtTrans,^17^ a pretrained protein language model (PLM) which provides a state-of-the-art distributed representation for proteins. In particular, we downloaded precomputed ProtT5 embeddings from UniProt https://www.uniprot.org/help/embeddings. Each protein sequence was represented by a real value vector of 1024 components. Only for 1734 proteins, we found the ProtTrans representation. The small molecules included in our analysis were represented by SMILES strings with lengths ranging from 24 to 95 characters. For the sake of simplicity, chlorine and bromine atoms, denoted by “Cl” and “Br”, respectively, were tokenized by a single character (“D” and “E”, respectively). Furthermore, we added two additional characters to each SMILES string, “BoS” (“$”) and “EoS” (“∼”), to denote the beginning and the end of the string, respectively. Finally, each molecule was padded with a unique character (“€”) for reaching the longest SMILES string. Our final alphabet was composed of 33 characters. Each character was represented as a one-hot vector composed of 33 components. Using this representation, each SMILES was encoded as a binary matrix of dimension 97 × 33. Our final data set *D*_100nM_ was composed of 336,567 (*protein*, *ligand*) pairs with 1734 unique proteins and 221,759 unique ligands.

With the aim of building training and test sets used for training and testing our models, we adopted a widely accepted splitting strategy which is particularly relevant to tackle the unseen target cases.^16,20,27,28^ We randomly split the *D*_100nM_ data set and generated three different training (TR) and test (TE) sets with two-thirds of the data in the training set and one-third in the test set. Moreover, in each pair (TR_*i*_,TE_*i*_) for *i* = 1, 2, and 3 we required the sequence similarity between the proteins in training and those in test sets to be less than 80%. To assess sequence similarities between protein pairs we used the Needleman-Wunsch global alignment algorithm from Biopython package.^29^ The distribution of pairwise sequence similarities for the first split is depicted in Figure 1. The distributions of the similarities for the other splits are reported in Figure S1 in the Supporting Information (SI). Notably, the majority of the sequences in TR1 have a similarity less than 40% with respect to those belonging to TE1 (Figure 1A). The same consideration holds true for protein pairs within TR1 (Figure 1B). Importantly, if the model *M*_*i*_ is trained on TR_*i*_, all of the proteins in TE_*i*_ are *unknown* to the model. The rationale of the adopted splitting strategy is that, when we exclude all of the (protein,ligand) pairs relative to a protein from the training set and test the model on this unseen protein, we may assess the performance of Prot2Drug on poorly studied proteins for which we know few or no binders.

**Figure 1:**
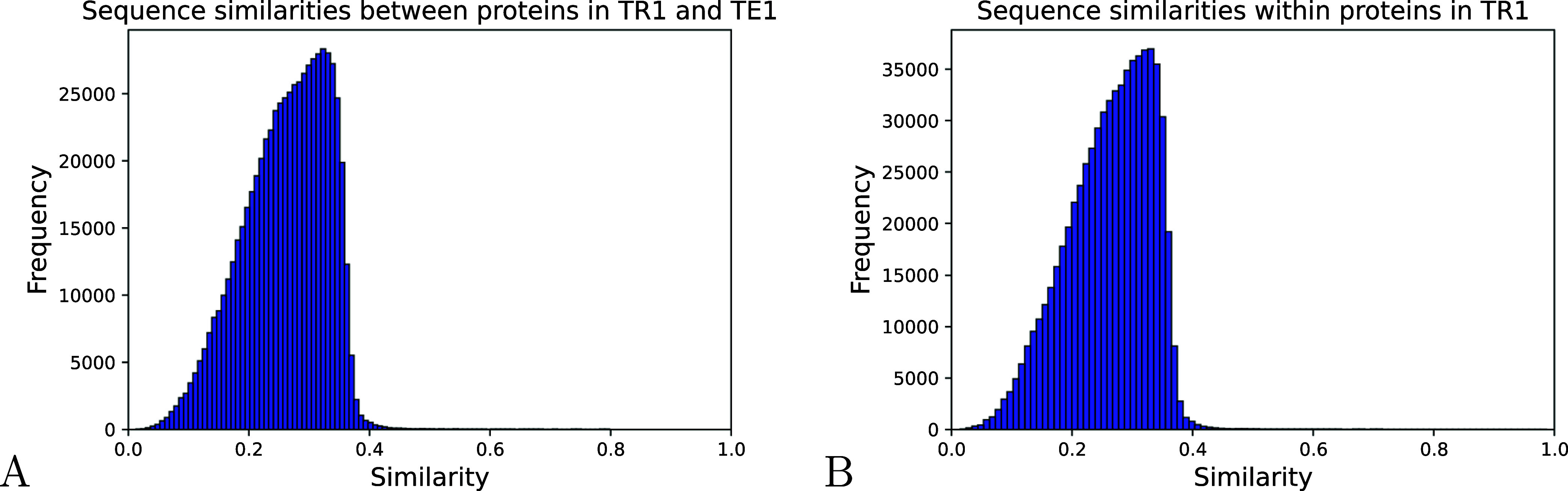
Distribution of sequence similarities among protein pairs. (A) Sequence similarities between proteins belonging to TR1 and TE1; (B) sequence similarities of proteins within TR1.

### Quality Metrics

The quality of the generated molecules were assessed by evaluating metrics commonly employed in the early stages of drug discovery process: (i) the percentage of chemically valid molecules (validity); (ii) the percentage of unique generated molecules calculated over the valid molecules (unicity); (iii) the percentage of generated molecules associated with the input protein not found in the training set (novelty), calculated over the unique generated molecules; (iv) the average quantitative estimate of drug-likeness (QED) score;^30^ (v) the average synthetic accessibility (SA) score;^31^ (vi) the internal diversity (ID)^32^ defined as the mean over the Tanimoto distances between each molecule and all of the others belonging to the same set; (vii) the average maximum Tanimoto similarity (MaxSim),^33^ calculated using circular Morgan fingerprints with a radius of 2,^34^ considering for each generated molecule the most similar molecule in the reference set (training or test) active on the target protein sequence; (viii) the average octanol–water partition coefficient indicative of molecular lipophilicity (log *P*); (ix) the molecular weight (*M*_W_); and (x) the percentage of generated compounds devoid of pan-assay interference compounds (PAINS), namely, structural alerts known to produce false positives in in vitro assays.^35^ All metrics were evaluated using the RDKit package^36^ and in-house Python scripts.

### Docking

The crystal structures of human Sigma-1 receptor (PDB code: 5HK1 ^37^) and Progesterone Receptor Ligand Binding Domain (PDB code: 1SQN^38^) were retrieved and pretreated using the Protein Preparation Workflow tool available from the Schrödinger suite 2024–1.^39^ The pretreatment protocol enables the addition of missing hydrogen atoms, reconstruction of incomplete side chains, assignment of ionization states at physiological pH, correction of the orientation of any misoriented groups, removal of water molecules, optimization of the hydrogen bond network, and execution of a force field-based minimization (OPLS-4)^40^ of the 3D protein structure. All of the ligands were prepared using LigPrep^39^ allowing to build the 3D structures, desalt, and generate all tautomers and ionization states at a pH value of 7.0 ± 2.0. For each receptor, a cubic grid was generated on the centroid of the cognate ligand obtaining an inner box of 10 Å × 10 Å × 10 Å and an outer box having an edge of 26.6 Å for Sigma-1 receptor and 23.3 Å for Progesterone Receptor Ligand Binding Domain, respectively. Standard docking simulations were performed using Grid-based ligand docking with energetics (GLIDE),^41^ available from the Schrödinger Suite 2024–1 as software program. During the docking simulations, the receptor protein was kept rigid, while the ligands were allowed full conformational flexibility. All simulations were conducted using the default force field OPLS_2005 ^42^ and the standard precision docking (SP) protocol.

### Prot2Drug Model

Prot2Drug is a generative model that is trained by using (protein, ligand) pairs (*P*, *l*) where the ligand *l* binds the protein *P* with an affinity <100 nM. During the training phase, Prot2Drug learns an estimate of the conditional probability density function *f*(*l*|*P*) for each protein belonging to the training set. During the testing phase, Prot2Drug samples this probability function for an input protein *P* by using the procedure described below and generates molecules *l*_1_, *l*_2_, ···, and *l*_*n*_ as observations of the random variable *l* given *P*. Our deep generative model makes use of a decoder transformer (see Figure 2) as described in the original paper^15^ with a stack of *N* = 2 identical layers, *h* = 32 heads and a model dimension *d*_*m*_ = 1024 equal to the size of adopted protein embedding. Due to the lack of the encoder, our model uses the multi-head attention mechanism in a slightly different way. In “encoder-decoder attention” layers, the queries come from the previous decoder layer, and the keys and values are computed by using the distributed representation of the input protein. In detail, let (*x*, *S*) be an input pair where $x\in R^{d_{m}}$ is the distributed representation of the protein and $S\in R^{L\times s}$ is the matrix representing the ligand, where *L* is the SMILES length and *s* the input size, i.e., the size of the alphabet. Let $E\in R^{s\times d_{m}}$ be an embedding matrix to be learned during training and $P\in R^{L\times d_{m}}$ be a constant positional encoding matrix as defined in^15^. Then, an input to the bottom layer of the decoder is

Y=SE+P

with $Y\in R^{L\times d_{m}}$ . In order to illustrate as the decoder used the protein distributed representation *x* and the ligand representation *Y*, let us consider the weight matrixes $W^{Q}\in R^{d_{m}\times d_{k}}$ , $W^{K}\in R^{d_{m}\times d_{k}}$ , and $W^{V}\in R^{d_{m}\times d_{k}}$ used for computing the query, key and value matrixes, respectively, where $d_{k}=\frac{d_{m}}{h}$ . The model used *Y* for evaluating the query matrix as follows:

$$Q=YW^{Q}\;\mathrm{with}\;\mathrm{sizes}\;\left(L\times d_{m}\right),\left(d_{m}\times d_{k}\right)$$

and the vector $x\in R^{d_{m}}$ for evaluating key and value matrixes

$$K=xW^{K}\;\mathrm{with}\;\mathrm{sizes}\;\left(1\times d_{m}\right),\left(d_{m}\times d_{k}\right)$$

$$V=xW^{V}\;\mathrm{with}\;\mathrm{sizes}\;\left(1\times d_{m}\right),\left(d_{m}\times d_{k}\right)$$

where $Q\in R^{L\times d_{k}}$ , $K\in R^{1\times d_{k}}$ , and $V\in R^{1\times d_{k}}$ . So the attention matrix *Z* is defined as

$$Z=\mathrm{attention}\left(Q,K,V\right)=\mathrm{softmax}\left(\frac{QK^{T}}{\sqrt{d_{k}}}\right)V$$

where $Z\in R^{L\times d_{k}}$ . The multi-head attention mechanism followed the original paper.^15^ For each attention module, the decoder evaluated an attention matrix $Z_{i}\in R^{L\times d_{k}}$

$$Z_{i}=\mathrm{attention}\left(Q_{i},K_{i},V_{i}\right),i=1,2,\cdot \cdot \cdot ,h$$

and built a matrix *Z* as

$$Z=\mathrm{concat}\left(Z_{1},Z_{2},\cdot \cdot \cdot ,Z_{h}\right)W^{O}$$

obtained concatenating all of the attention heads *Z*_1_, *Z*_2_, ···, *Z*_*h*_. Note that concat­(*Z*_1_, *Z*_2_, ···, *Z*_*h*_) is a matrix of size *L* × *d*_*m*_ because *d*_*m*_ = *hd*_*k*_ and then *W* ^*O*^ is a matrix of size *d*_*m*_ × *d*_*m*_. Notice that the multi-head attention module produces an output matrix *Z* which has the same size *L* × *d*_*m*_ as the input matrix *Y*. In this way, the self-attention layers in the decoder remained unchanged.

**Figure 2:**
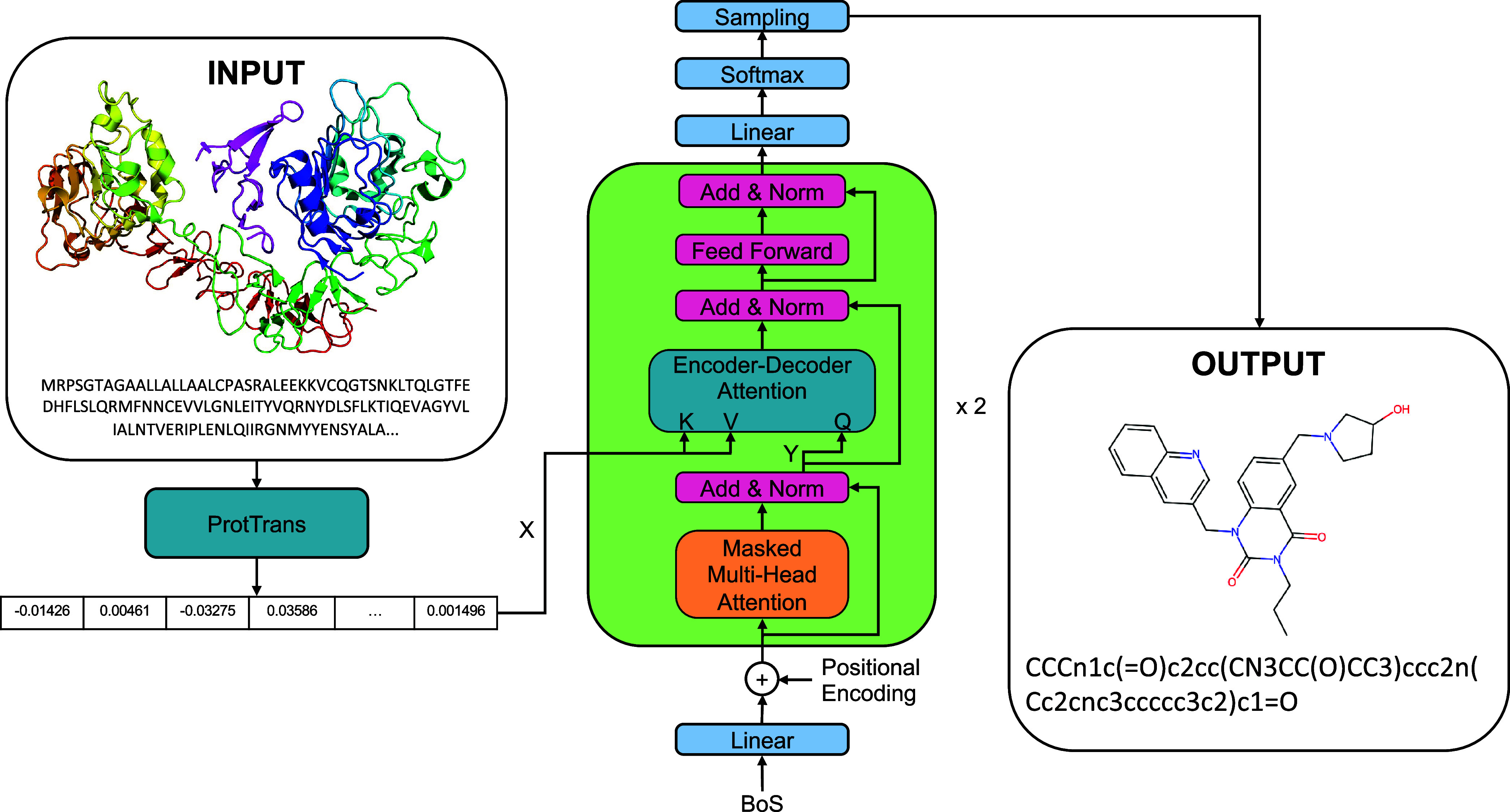
Architecture of the Prot2Drug generative model.

### Sequence Generation

After training, for a given distributed representation *x* of a query protein sequence, the model generated a sequence of characters (*c*_1_, *c*_2_, ···) starting from the special character “BoS” (“$”) and ending when the special character “EoS” (“∼”) was generated. The decoder is an autoregressive model which makes use of the previously generated symbols as input for generating the next one. Let us suppose the decoder generated *k* – 1 characters and let *p*_*k*_ = softmax­(*y*_*k*_) be the probability density function of the next character and *y*_*k*_ the decoder output. For generating the character *c*_*k*_ we made use of a well-known result in probability theory (see ref 43, page 139), according to which if *z* is a random variable with distribution *P* then the random variable *r* = *P*(*z*) is uniformly distributed in [0, 1]. So, we generated a random number *r* uniformly distributed in [0, 1] and determined the next character *c*_*k*_ as the character associated with the index *i* such that *r* = ∑_*j* = 1_^*i*^ *p*_*k*_(*j*).

### Training and Implementation

The model was trained by using a high-performance computing (HPC) architecture. In detail, this cluster was composed of 73 nodes POWER SYSTEM AC922 produced by IBM company. Each computing node was composed of 4 GPUs NVIDIA Tesla V100 with a dedicated memory of 32GB each, 2 processors POWER9 of 16 cores@3.3 GHz, 16 DDR4 RAM of 64 GB, and 2 hard disks SSD of 1.92 TB. The proposed model was trained by using four nodes, two GPUs per node, and a distributed data parallel strategy. The code was developed in Python v 3.9. Other auxiliary python-based packages were installed, such as pytorch (version 2.0.1), rdkit (version 2021.03.5), torchvision (version 0.15.2), and so on. The NVIDIA collective communication library (NCCL) was used as multi-GPU and multinode communication primitives by the pythorch modules. The hyperparameters of the model were set to their default values. The models were trained for 100 epochs using a batch size of 200. The learning rate was kept fixed to 0.001. The Adam optimizer was adopted as the optimization technique.

### Drug–Target Interaction Analysis

With the aim of assessing the performances of our model to generate new molecules binding a specific protein target, we used ConPLex^20^ a high-throughput computational prediction method of drug–target interactions (DTIs). To this end, we first evaluated ConPLex’s performances globally by analyzing the predicted DTI scores on the whole set of known interactions in our data set *D*_100nM_. In particular, for each of the 1734 proteins, we considered true positive (TP) pairs (*A*, *l*_*A*_) formed by protein target *A* and all its known ligands *l*_*A*_ in *D*_100nM_. Moreover, for the same protein target *A*, we formed an equal number of true negative (TN) pairs (*A*, *l̅*_*A*_) composed of the same protein target and ligands randomly drawn from *D*_100nM_ binding protein targets different from *A*. A total of 673134 DTI pairs were assessed. Our statistical analysis showed that the ConPLex scores distinguished between TP and TN interaction pairs (Figure 3A,B). In fact, the one-sided, nonparametric Mann–Whitney test (AUC = 0.61, *P* < 1.0 × 10^–360^) showed that the DTI scores evaluated on TP pairs were greater than on TN ones. Moreover, by using the ConPLex threshold of 0.923 recommended for high-precision screening, we found that the set of top-ranked drug interaction scores (those having ConPlex score ≥ 0.923) was enriched (one-sided exact Fisher’s test *P* < 1.0 × 10^–360^) of TP pairs (1475) with respect to the TN pairs (21) on a total of 673,134 DTI scores. In order to test on which proteins ConPlex most accurately predicted drug–target interactions, we repeated the previous analysis on each protein separately. In particular, for each protein, we evaluated ConPLex scores on TP (protein, ligand) pairs and on an equal number of TN pairs. To this end, we measured the AUC values and performed a one-sided Mann–Whitney test for assessing the differences between the distributions of the scores in the 2 classes. The histograms of the obtained AUC values and *p*-values are depicted in Figure 3C,3D, respectively. We found 485 proteins with statistically significant AUC values with *P* = 1.37 × 10^–02^ and false discovery rate^44^ FDR = 5%. In conclusion, these findings indicate that ConPLex produced accurate predictions of interaction and that in general, the TP interactions had ConPLex scores greater than TN ones.

**Figure 3:**
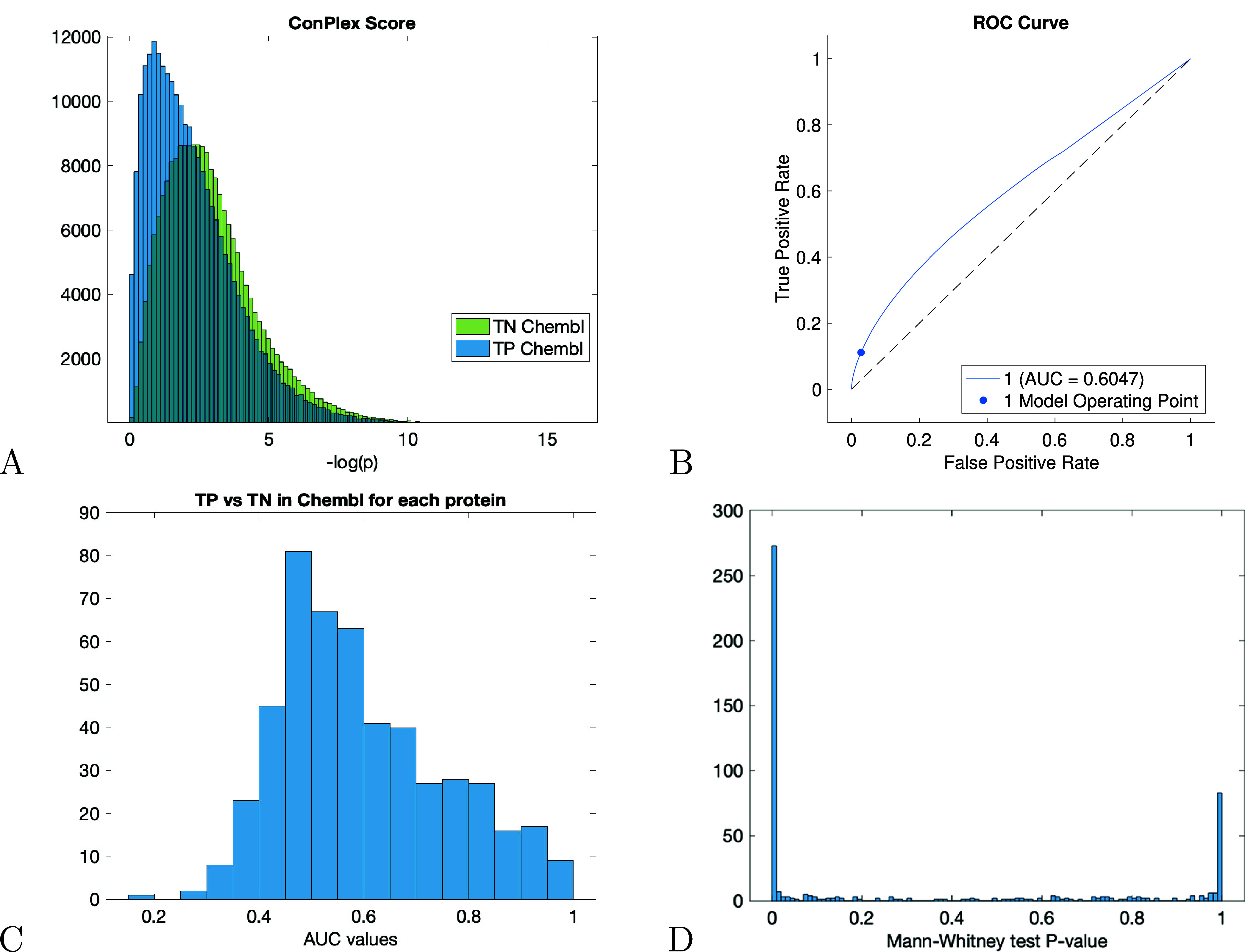
Ground-truth on *D*_100nM_. (A) Histograms of the DTI scores predicted by ConPLex on TP and TN pairs in *D*_100nM_ and (B) the corresponding ROC curve. (C) Histogram of AUC values associated with each protein target assessed by using the ConPLex scores in the comparison TP and TN protein–ligands pairs. (D) Histogram of the *p*-values of the one-sided Mann–Whitney test.

## Results

As mentioned above, Prot2Drug is a deep learning model for de novo drug design that generates novel potential ligands of a protein target by relying on its sequence embedding only. The integration of ProtTrans embeddings with transformer models and the knowledge of thousands of protein–ligand interactions from the ChEMBL database enable us to accurately capture the lexicon of proteins to bind specific small molecules. Starting from the sequence embedding of a protein, Prot2Drug learns to design the SMILES of its new potential ligands from scratch (see the Methods section). In detail, we trained Prot2Drug on three different training sets obtaining three models *M*_1_, *M*_2_, and *M*_3_ (see the Methods section). We used these models for generating molecules by using ProtTrans distributed representation both for never seen before proteins belonging to the respective test set (TE), and for known proteins belonging to the training set (TR). Each model generated 1000 molecules for protein in both scenarios. We used RDkit for checking the validity of all the generated SMILES strings. As reported in Table 1, the values of validity, unicity, and novelty are nearly consistent across the different training and test sets, although the novelty tends to be higher in the test sets compared to the training sets. This difference arises from the fact that the proteins within the test sets are unknown to the model. Moreover, they are comparable with the values reported by other recently developed generative models such as ORGAN,^45^ LatentGAN,^46^ and DeepTarget^16^ (see Table 1) which exploit novel and different deep learning architectures for drug de novo design. In particular, in the case of validity, Prot2Drug showed a slightly inferior performance than DeepTarget. Notice that the values of validity exhibited by Prot2Drug do not indicate a shortcoming of the proposed chemical language model. In fact, as pointed out by Skinnider,^47^ the generation of a few invalid SMILES is a desirable property for a generative model because it provides an intrinsic mechanism to identify and remove low-quality samples from the model output. Moreover, it is an indicator of the model’s ability to explore unseen regions of the chemical space. Furthermore, generating only valid molecules is not our ultimate goal; a few invalid SMILES can be removed a posteriori by a fast and simple algorithm in RDKit. Compared with works that only focused on effective molecules, our study also assessed the predicted binding power of molecules generated with the target protein using ConPLex and docking scores and the ability of the model to exactly reproduce the known data active compounds not encountered during training. Henceforth, with the term “new molecule” we mean a generated and valid molecule that is absent in the training set.

**Table 1:** Percentages of Valid, Unique, and Novel Molecules Generated by the Three Models by Using Proteins in Different Training and Test Sets (TR Indicates Training Set and TE Test Set), as well as by Three Recently Developed Generative Models

	*M_1_*		*M*_2_		*M*_3_				
	TR1	TE1	TR2	TE2	TR3	TE3	ORGAN^45^	LatentGAN^46^	DeepTarget^16^
validity	74%	69%	79%	74%	72%	67%	64%	71%	80%
unicity	79%	90%	73%	88%	77%	88%	99%	99%	99%
novelty	95%	98%	94%	97%	96%	98%	99%	99%	99%

### Physicochemical Properties of New Molecules

The new molecules reproduced relevant physicochemical properties of the ligands present in the data set *D*_100nM_. We first assessed the properties of the molecules generated by the model *M*_1_ by using the ProtTrans representations of proteins belonging to TR1 as input. To this end, we applied numerous quality metrics generally employed to evaluate the performances of generative models for de novo design^11,25,26,48^ assessing the molecular properties of the generated molecules and then averaging them on all molecules generated from single proteins (see the Methods section). The generated molecules were both druglike and easily synthesizable, as evident from the distributions of the average values of quantitative estimation of drug-likeness (QED > 0.5) and synthetic accessibility (SA < 3.5) (Table 2 and Figure 4A,B respectively) for almost all of the new molecules.^30,31^ Surprisingly, the values of QED and SA evaluated on the new molecules are greater, respectively, less, than the ones measured on the ligands in *D*_100nM_ (*P* = 4.73 × 10^–06^ and *P* = 1.49 × 10^–09^ one-sided Mann–Whitney test) for the same protein targets. Moreover, the average QED scores measured on the new ligands generated by *M*_1_ by using the protein targets belonging to TR1 were strongly correlated to the scores measured on the ligands in *D*_100nM_ for the same targets (Pearson correlation coefficient PCC = 0.90, SI Appendix Figure S8A). Only protein targets with at least 20 ligands in *D*_100nM_ were considered. The same considerations hold true for the average SA scores (PCC = 0.90, SI Appendix Figure S9A). The plots clearly highlight that the generated molecules reproduced the key features of the *D*_100nM_ molecules in terms of drug-likeness and synthetic accessibility, confirming our algorithm’s ability to sample the chemical space associated with the input proteins. We also computed log *P* and molecular weight (*M*_W_) for the generated sets (TR1, TE1, TR2, TE2, TR3, and TE3). As reported in Table 2, the values obtained align with those expected for drug candidates, with an average *M*_W_ slightly above 400 Da and log *P* ranging between 3.54 ± 1.45 and 3.75 ± 1.51. Notably, these values are consistent with those derived from the training set molecules (*D*_100nM_), which exhibit average *M*_W_ and log *P* values of 447.42 ± 95.26 and 3.36 ± 1.61, respectively. Detailed distributions of each property are in Figures S2–S7 in the Supporting Information. The same analysis was performed on the new molecules generated by the model *M*_1_ by using protein sequences unseen by the model during learning because they belong to the test set TE1. Also in this case, the generated molecules exhibited good druglikeness and synthetic accessibility (Table 2 and Figure 4E,F, respectively) with average values of QED and SA measured on the new molecules correlated to those measured on *D*_100nM_ for the same targets (SI Appendix PCC = 0.54 (*P* = 1.54 × 10^–21^) in Figure S8B and PCC = 0.46 (*P* = 1.24 × 10^–15^) in Figure S9B for QED and SA, respectively). The observed decrease in the correlation values is likely a consequence of the generation of molecules for novel protein sequences not used in the training process. Moreover, also for the test set TE1, the generated molecules have log *P* values and molecular weights similar to molecules in *D*_100nM_ (Figures S6B and S7B in the Supporting Information). Finally, to further evaluate the performance of Prot2Drug, it was compared with other state-of-the-art approaches for target-specific molecular generation on the values of log P, QED, and SA (Table 3). For this comparison, we considered only methods more similar to Prot2Drug incorporating information on target proteins in the learning phase. In particular, we analyzed the mean values of these physicochemical properties for molecules generated by the following methods: the original transformer with beam search adopted by Greschnikova,^14^ LiGANN,^49^ SBMolGen,^50^ SBDD-3D,^51^ and Alphadrug.^52^ Two versions of AlphaDrug were considered: AlphaDrug (freq) refers to the molecular generation by the execution of Monte Carlo Tree Search with random selection of the next symbol according to the frequency of visited times of simulations, and Alphadrug (max) indicates the MonteCarlo Tree Search with the selection of the next symbol with the maximum visited times of simulations. For each method, the values of the physicochemical properties were computed on 10 generated ligand candidates for each testing protein. For fair comparisons, we also used Prot2Drug to generate 10 molecules for each testing protein. The results in Table 3 indicate that the produced molecules by Prot2Drug, LIGANN, and SBMolGen have comparable QED and SA scores, whereas Greschnikova and AlphaDrug have slightly inferior values of QED and SBDD-3D has a higher value of SA. All methods produce molecules with log *P* in a suitable range for drugs.

**Table 2:** Physicochemical Properties of New Molecules Generated by Prot2Drug

set name	ID^a^	QED^b^	SA^c^	MaxSim^d^	nPAINS (%)^e^	log *P* ^f^	*M*_W_ ^g^
TR1	0.78	0.54 ± 0.11	2.89 ± 0.33	0.38 ± 0.18	96.75	3.71 ± 1.47	425.89 ± 82.92
TE1	0.80	0.56 ± 0.18	2.82 ± 0.56	0.27 ± 0.08	97.03	3.75 ± 1.51	418.49 ± 83.37
TR2	0.78	0.55 ± 0.11	2.84 ± 0.36	0.39 ± 0.18	96.26	3.64 ± 1.44	418.17 ± 82.83
TE2	0.80	0.57 ± 0.18	2.75 ± 0.56	0.28 ± 0.08	96.46	3.63 ± 1.46	405.93 ± 82.41
TR3	0.78	0.55 ± 0.11	2.89 ± 0.38	0.38 ± 0.18	96.54	3.54 ± 1.45	412.56 ± 82.81
TE3	0.79	0.57 ± 0.18	2.80 ± 0.58	0.29 ± 0.09	96.76	3.56 ± 1.46	404.51 ± 82.71
*D*_100nM_	0.63	0.47 ± 0.16	3.23 ± 0.70	n.c.	96.77	3.36 ± 1.61	447.42 ± 95.26

a Internal diversity.

b Quantitative estimation of drug-likeness.

c Synthetic accessibility.

d Maximum similarity.

e Molecules predicted not to be PAINS.

f Log *P*.

g Molecular Weight (Da).

**Figure 4:**
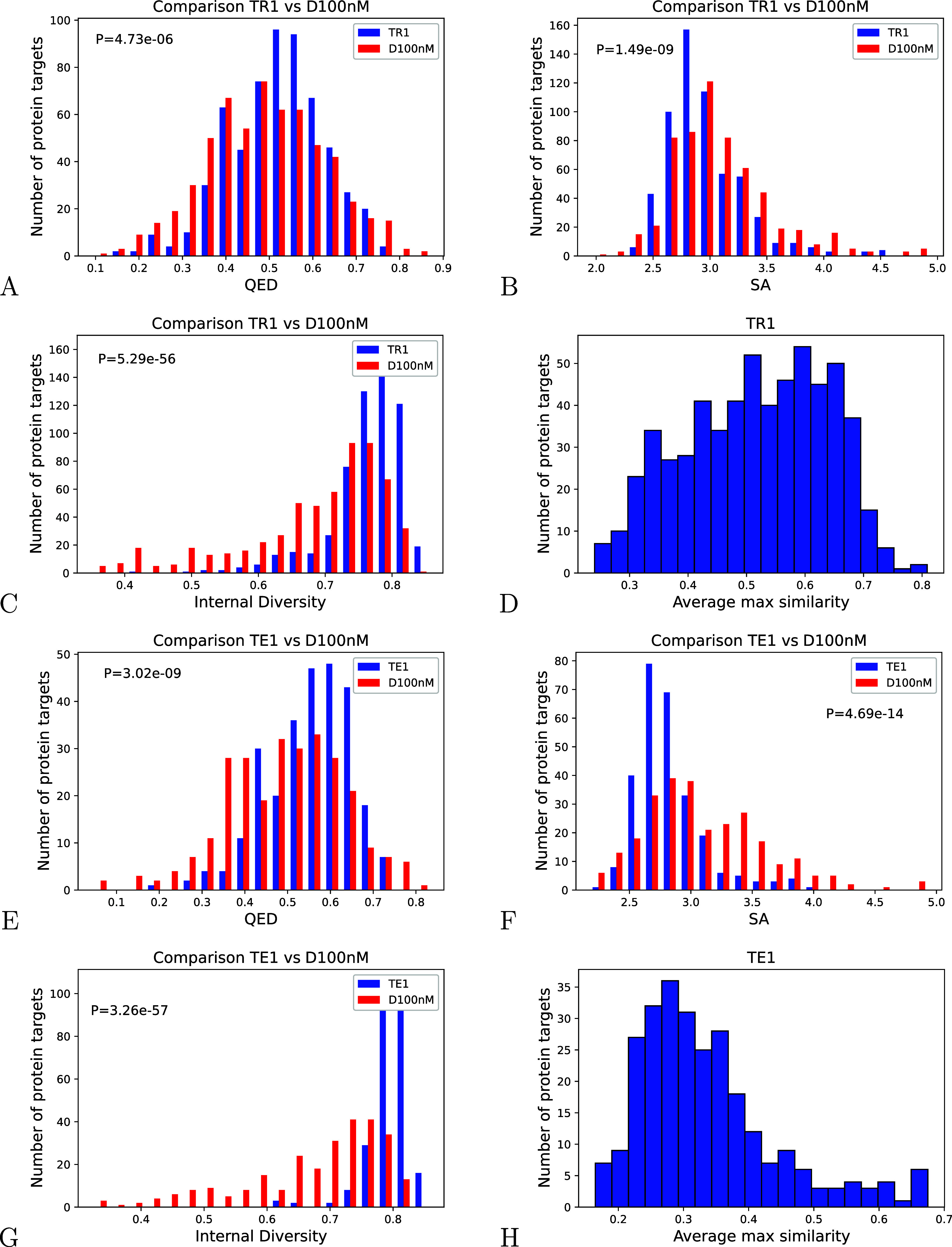
(A, B, C, D) Histograms of QED, SA, ID and MaxSim assessed on new molecules generated by using proteins belonging to TR1, respectively. (E, F, G, H) Histograms of QED, SA, ID and MaxSim assessed on new molecules generated by using proteins belonging to TE1, respectively.

**Table 3:** Comparison of log *P*, QED, and SA Average Values with Respect to Other Generative Models

model	log *P*	QED	SA
LiGANN^49^	2.9	0.6	3.0
SBMolGen^50^	2.6	0.7	2.8
SBDD-3D^51^	1.5	0.6	4.0
Grechishnikova^14^	3.8	0.5	2.8
AlphaDrug (freq)^52^	4.9	0.4	2.9
AlphaDrug (max)^52^	5.2	0.4	2.7
Prot2Drug	3.6	0.6	2.8

#### Similarity Analysis

The analysis of the distributions of the Internal Diversity having average values greater than 0.78 points out the great diversity of the generated ligands compared to the known ligands in *D*_100nM_ (Table 2 and Figure 4C,G). In particular, the Internal Diversity measured on the new molecules is greater than the one relative to the compounds in *D*_100nM_ (*P* = 5.29 × 10^–56^). The large Internal Diversity values demonstrate that the algorithm is able to generate molecules spanning a large chemical space, as requested in the context of de novo drug design.^2^ Moreover, the maximum Tanimoto similarity values less than 0.39 highlight the model’s ability to generate molecules with a good degree of novelty compared to those in *D*_100nM_ for the considered protein targets (Table 2 and Figure 4D). In particular, the analysis of the average maximum Tanimoto similarity, assessed on the new molecules generated by using protein targets in test sets with respect to their known ligands (0.27, 0.28, and 0.29 on TE1, TE2, and TE3, respectively), indicates that Prot2Drug is able to generate new molecules with a higher degree of novelty for never seen before protein targets (Table 2 and Figure 4H). Taken together, these results highlight the algorithm’s potential to generate valid drug candidates with a large chemical diversity (SI Appendix Figure S5) owning good druglikeness and novelty (SI Appendix Figure S2) for use even with entirely new protein targets for which no active ligands are known. A complete description of the physicochemical properties of the new molecules generated by the models *M*_2_ and *M*_3_ is provided in the SI Appendix (Figures S2–S9).

### Testing the Predicted Binding Affinity between Generated Compounds and Target Proteins

In this section, we show that the new molecules *l*_*A*_ generated by Prot2Drug by using the embedding of protein targets *A* are promising candidates to bind the target *A*. To this end, we did not directly compare the generated molecules with known binders of the target *A* belonging to training or test sets by structural similarity measures. Instead, we used ConPLex,^20^ a recent open source, sequence-based drug–target interaction (DTI) prediction method which provides state-of-the-art performances. In particular, we assessed the binding scores predicted by ConPLex on protein–ligand pairs (*A*, *l*_*A*_) composed of protein *A* and the new molecules generated from *A* and compared them with the scores predicted on random pairs (*A*, *r*_*A*_) composed of the same targets *A* and random molecules *r*_*A*_. Notice that with the term “new molecule” we mean a generated, valid, and unique molecule not present in the training set. Formally, for each protein target *A*, we composed the following sets of protein–ligand pairs:POS_*G*_ = {(*A*, *l*_*A*_)|*l*_*A*_ was generated
by Prot2Drug
by using the protein target *A*};NEG_*G*_ = {(*A*, *r*_*A*_)|*r*_*A*_ was randomly drawn from the
new molecules generated by Prot2Drug by using a protein target different
from *A* };NEG_*D*_100nM__ = {(*A*, *r*_*A*_)|*r*_*A*_ was randomly
drawn from the molecules in *D*_100nM_ binding
a protein target different from *A*};TRUE_POS = {(*A*, *k*_*A*_)|*k*_*A*_ was a known binder of the protein target *A*}.


In our analysis, for each protein, the sets involved in the pairwise comparisons were composed of the same number of pairs. We evaluated the binding scores predicted by ConPLex for each set. Moreover, for testing the hypothesis that the binding scores relative to positive pairs were greater than the ones relative to negative pairs, we evaluated for each protein the area under the ROC curve (AUC) and the *p*-value of the one-sided Mann–Whitney test and counted the number of protein targets having a statistically significant *p*-value among the considered targets. The problem of multiple comparisons was addressed by using the False Discovery Rate (FDR).^44^

Let us start by considering the case in which the new molecules were generated by Prot2Drug using *known* protein targets belonging to the training set TR1. In this case, the proteins used for the generation of the new ligands were known to Prot2Drug because seen during the training phase.

Comparison a. Comparing the sets POS_*G*_ versus NEG_*G*_, we found 586 on 1156 protein targets with *P* ≤ 2.48 × 10^–02^ and false discovery rate^44^ FDR = 5% (see Figure 5A) indicating that for more than half of the analyzed protein targets, the generated molecules exhibited binding scores higher than random molecules. Moreover, we compare these AUC values with the ones evaluated on *D*_100nM_ for the same protein targets (see the Methods section) and measured a correlation of PCC = 0.82 (*P* = 2 × 10^–175^) (see Figure 5B).

**Figure 5:**
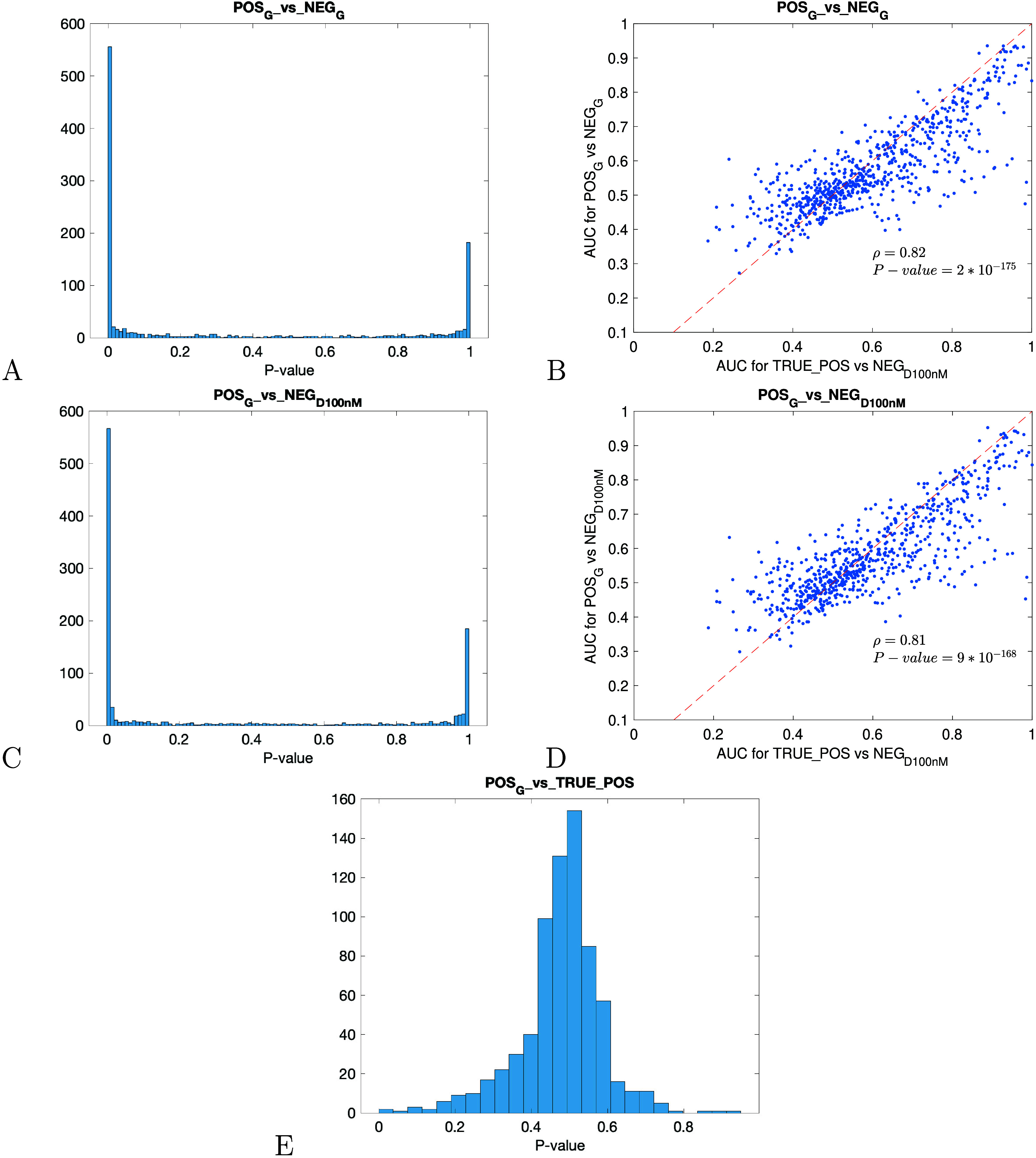
Statistical assessment of the AUC values evaluated using the new molecules generated using the protein targets in the training set TR1. (A, C) *P*-value histograms of the Mann–Whitney test in the comparisons a and b, respectively. (B, D) Scatter plots of the AUC values: *x*-axis represents the values measured by using the known protein–ligands interactions in *D*_100nM_ for each protein and *y*-axis represents the values measured in Comparisons a and b, respectively. (E) Histogram of the AUC values evaluated in Comparison c.

Comparison b. Comparing the sets POS_*G*_ versus NEG_*D*_100nM__, we found 606 proteins with *P* ≤ 2.56 × 10^–02^ at FDR = 5% confirming the previous result (see Figure 5C). Also in this case, the AUC values correlated with the ones evaluated on *D*_100nM_ for the same protein targets (PCC = 0.81 with *P* = 9 × 10^–168^, Figure 5D).

Comparison c. Comparing the sets POS_*G*_ versus TRUE_POS, and considering the set of protein targets having a number of known ligands ≥10 in *D*_100nM_, we found a mean value of AUC and standard deviation of 0.48 ± 0.11 indicating that new molecules and known ligands had comparable predicted binding scores assessed on the same protein targets (see Figure 5E).

Analogue comparisons were performed in the case in which the new molecules were generated by Prot2Drug using *unknown* protein targets belonging to the test set TE1. In this case, the proteins used for the generation of the new ligands were unknown to Prot2Drug because not seen during the training phase.

Comparison d. Comparing the sets POS_*G*_ versus NEG_*G*_, we found 284 on 557 protein targets with statistically significant AUC values with *P* ≤ 2.32 × 10^–02^ at FDR = 5% (see Figure 6A). Also in this comparison, the AUC values were correlated with the ones evaluated on *D*_100nM_ for the same protein targets, although with a lower correlation value (PCC = 0.48 with *P* = 3 × 10^–20^, Figure 6B).

**Figure 6:**
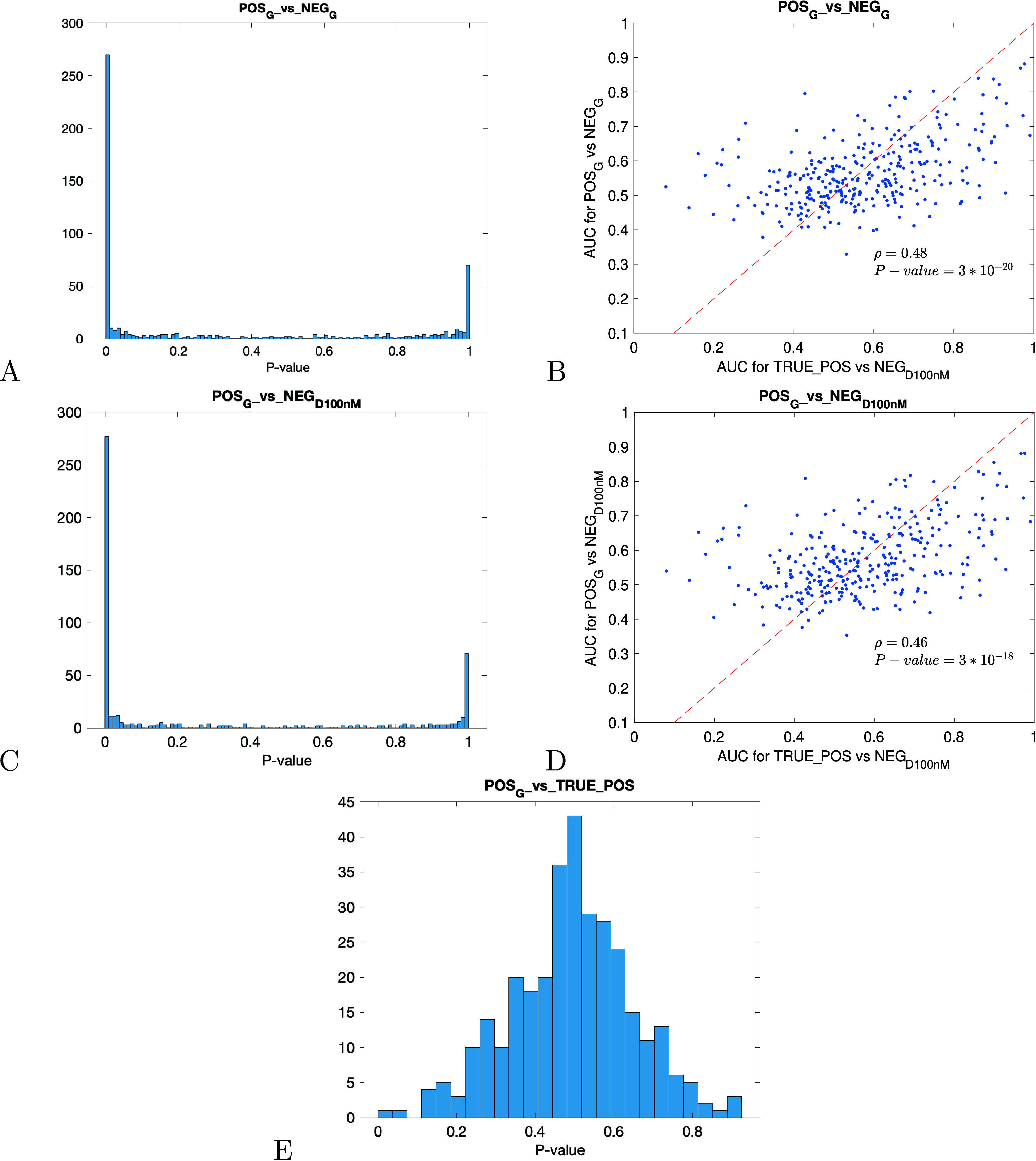
Statistical assessment of the AUC values evaluated using the new molecules generated using the protein targets in test set TE1. (A, C) *P*-value histograms of the Mann–Whitney test in Comparisons d and e, respectively. (B, D) scatter plots of the AUC values: *x*-axis represents the values measured by using the known protein–ligands interactions in *D*_100nM_ for each protein and *y*-axis represents the values measured in Comparisons d and e, respectively. (E) Histogram of the AUC values evaluated in Comparison f.

Comparison e. Comparing the sets POS_*G*_ versus NEG_*D*_100nM__, we found 296 protein targets with *P* ≤ 2.61 × 10^–02^ at FDR = 5% (see Figure 6C). The AUC values were correlated with the ones evaluated on *D*_100nM_ for the same protein targets (PCC = 0.46 with *P* = 3 × 10^–18^, Figure 6D).

Comparison f. Comparing the sets POS_*G*_ versus TRUE_POS, and considering the set of protein targets having a number of known ligands ≥10 in *D*_100nM_, we found a mean value of AUC and standard deviation of 0.49 ± 0.15 confirming that new molecules and known ligands had comparable binding scores assessed on the same protein targets (Figure 6E).

A complete statistical analysis of the binding affinities involving compounds generated by Prot2Drug by using known protein targets belonging to training sets TR2 and TR3, as well as by using unknown proteins belonging to test sets TE2 and TE3, is described in the Supporting Information (Section 3 and Figures S10–S13). A list of newly generated molecules having a ConPLex score >0.923 is provided in SI Data Set S6 together with QED and SA values.

Moreover, a representative set of molecules generated by Prot2Drug using unseen proteins in TE1 and returning high ConPLex scores for different targets is shown in Figures 7 and 8. Notice that a pool of structures generated by Prot2Drug using proteins in TR1 is depicted in Figures 9 and 10. Taken together, all of these statistical assessments showed that the generated molecules were promising ligands as the returned predicted binding scores were comparable to those displayed by the known ligands used for training the model. Moreover, they provide a first proof-of-concept of our method.

**Figure 7:**
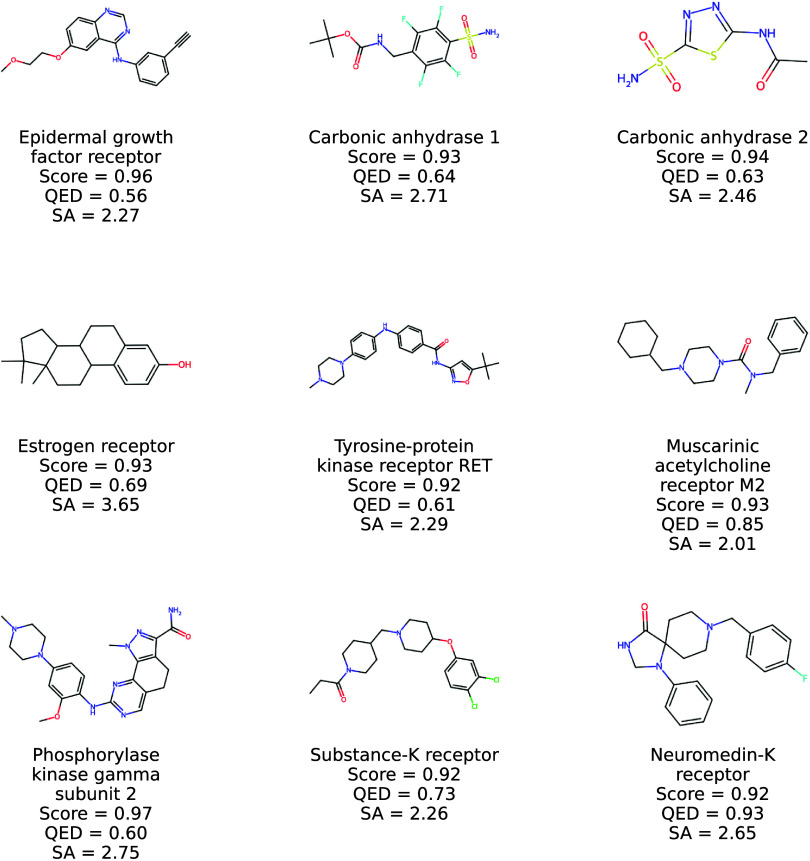
2D structures of new molecules generated by Prot2Drug by using protein targets in TE1 together with input proteins, ConPLex scores, and QED and SA values.

**Figure 8:**
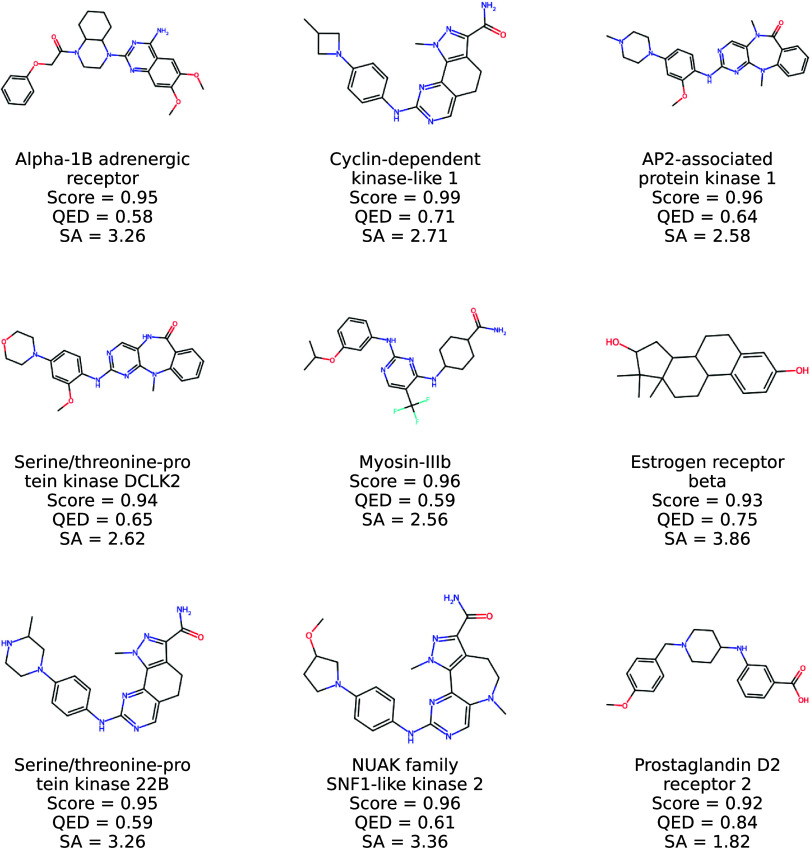
More 2D structures of new molecules generated by Prot2Drug by using protein targets in TE1 together with input proteins, ConPLex scores, and QED and SA values.

**Figure 9:**
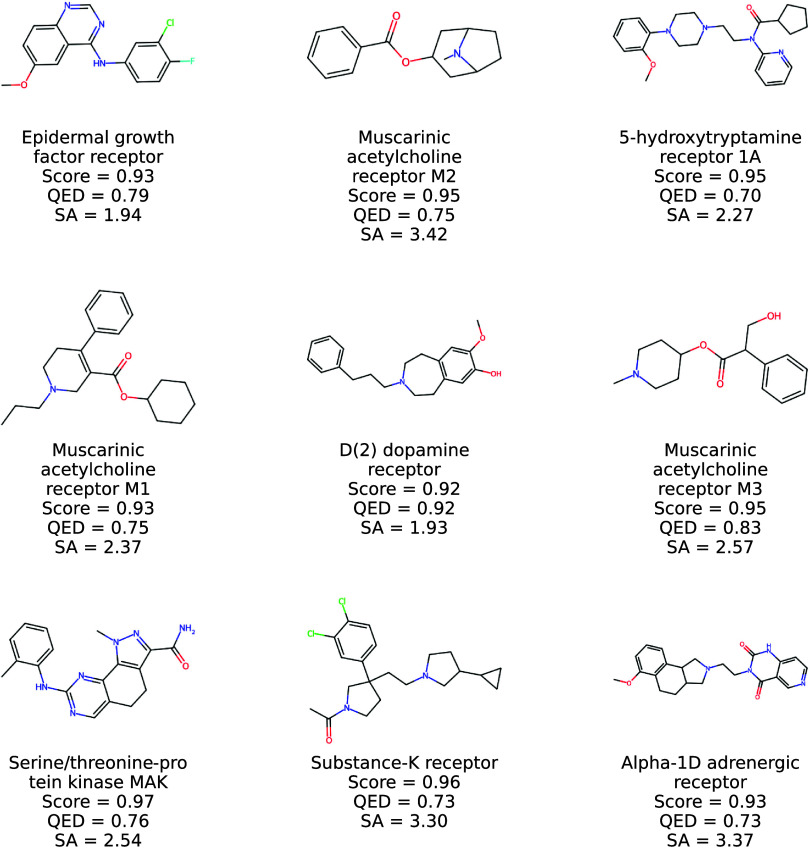
2D structures of new molecules generated by Prot2Drug by using protein targets in TR1 together with input proteins, ConPLex scores, and QED and SA values.

**Figure 10:**
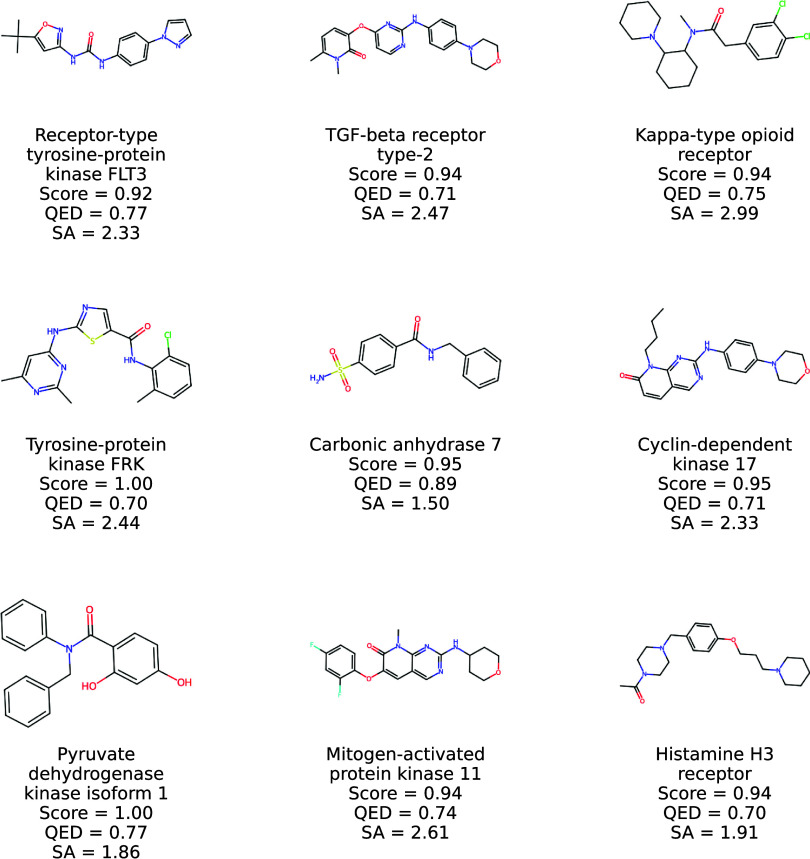
More 2D structures of new molecules generated by Prot2Drug by using protein targets in TR1 together with input proteins, ConPLex scores, and QED and SA values.

### Reproducibility of Known Ligands

According to Chen and Bajorath^21^ which indicated the ability of a generative model to exactly reproduce known active compounds not encountered during training as the most rigorous proof-of-concept criterion for the approach, we analyzed the ability of Prot2Drug to reproduce known active binders. Prot2Drug reproduced numerous known ligands not encountered during training, binding protein targets with a known affinity value <100 nM. In detail, let us consider the molecules generated by the model *M*_1_ by using unknown protein targets belonging to the test set TE1. For each of the 557 protein targets, the model generated 1000 molecules. Notice that, according to our experimental design, if a protein target belonged to the test set, that protein and all its ligands were unknown to the model. Prot2Drug reproduced 66 known compounds obtained intersecting the newly generated molecules with the ligands present in TE1 (see Table 4). Now there exist two relevant cases that we want to highlight. The first case is when the molecule *l_A_* generated by model *M*_1_ by using protein target *A* produces a known pair (*A*, *l*_*A*_) present in test set TE1. We call *l*_*A*_ a *proper* ligand because it is generated from the protein *A*. For example, among the 66 reproduced compounds generated by *M*_1_, 24 fell into this category producing 25 unique known pairs (*A*, *l*_*A*_) where *l*_*A*_ was generated by *A* (SI Data Set S1). The second case, that we call *repurposing*, is when the molecule *l*_*A*_ generated by the model *M*_1_ by using the protein *A* binds a different protein *B* producing a known pair (*B*, *l*_*A*_) present in test set TE1. In other words, the model starting from an unknown protein *A* generates a ligand *l*_*A*_ which binds a different protein *B*. We call this ligand a *repurposed* ligand because, generated from the protein *A*, binds a different target *B*. We denote this case with the triple (*A*, *l*_*A*_, *B*). By using the 66 reproduced compounds generated by the model *M*_1_, we found 82 triple of the type (*A*, *l*_*A*_, *B*) (SI Data Set S2).

**Table 4:** Number of Reproduced Known Ligands and Interactions Generated by the Three Models

	*M*_1_		*M*_2_		*M*_3_	
	TE1	TR1	TE2	TR2	TE3	TR3
reproduced ligands	66	172	175	303	172	352
reproduced interactions	25	0	58	0	63	0
repurposed interactions	82	244	264	631	308	822

Analogously, let us consider the new molecules generated by model *M*_1_ by using the known protein targets belonging to the training set TR1. Notice that if *A* is a protein target in TR1, all its known ligands in *D*_100nM_ were present in TR1. So, if *l*_*A*_ is a *new* molecule generated by *M*_1_ by using *A*, we have no information concerning the binding affinity of the (*A*, *l*_*A*_) pair. We found 172 new molecules generated by the model *M*_1_ and with these reproduced molecules we identified 244 triples (*A*, *l*_*A*_, *B*) in which the (*B*, *l*_*A*_) pairs were known protein–ligand pairs (SI Data Set S3). Table 4 reports the number of known compounds reproduced by the *M*_2_ and *M*_3_ models (see SI Data Sets S1, S2, S4, and S5). In conclusion, these experimental findings show that our models reproduced numerous known ligands with nanomolar affinity. These molecules bind the protein targets used for generating them (proper ligands) and bind different proteins (repurposing). Remarkably, the reproduction of proper ligands is particularly relevant because it makes us confident in the Prot2Drug’s ability to design new molecules binding the targets used for generating them. Moreover, the reproduction of ligands known to bind different proteins may help identify off-targets, which may result in adverse drug reaction or suggest new strategies for repurposing the compound. Among the reproduced compounds for repurposing, some involve proteins sharing many known binders, confirming that the ligands generated by Prot2Drug reproduce the knowledge present in the data and making us confident that they are good binders of the proteins used to generate them. On the other hand, a reproduced compound involving proteins that do not share any common ligand in ChEMBL is also a very important finding that should be analyzed in depth since this suggests ligands that could co-target proteins that have no common known ligand. Among the reproduced molecules, examples of proper ligands are some known ligands of the Epidermal growth factor receptor (EGFR) protein. The method reproduced proper ligands of EGFR as CHEMBL94061, CHEMBL3699587, CHEMBL301018, and CHEMBL3814257. It is worth mentioning that CHEMBL94061, CHEMBL3699587, and CHEMBL3814257 are molecules unseen during the learning phase since they bind only to EGFR that was in the test set and consequently all its ligands were not in the training set. CHEMBL301018 was generated both from EGFR and from the Receptor tyrosine-protein kinase erbB-2 (ERBB2) proteins when each one was in the test set, and it is a known binder for both of them. Taken together, these observations also make us confident in the specificity of the novel candidate ligands generated from Prot2Drug for EGFR and ERBB2 that are attractive targets for their role in cancer progression. EGFR and ERBB2 have been shown to be involved in many aspects of oncogenic progression, such as sustaining cell growth, enhancing resistance to cell death, and reprogramming metabolic networks and their involvement in metastasis is a relevant research theme in the past few years.^24^ Examples of repurposed interactions concern known ligands of EGFR that were generated by Prot2Drug from the ERBB2 sequence, suggesting investigating the interaction between ERBB2 and these ligands. These novel drug–protein pairs are consistent with current knowledge of the two proteins. EGFR and ERBB2 are both members of the EGFR family of tyrosine kinases also known as the HER or ErbB receptor family, which share 645 ligands at the threshold of 100 nM activity. The molecules with ID CHEMBL4208811, CHEMBL3759615, CHEMBL2437469, CHEMBL14699, CHEMBL541586 (or CHEMBL94431), CHEMBL540068 (or CHEMBL7917), and CHEMBL3758283 were generated from ERBB2 but are known binders only for EGFR. It is interesting to note that also these ligands, together with the protein ERBB2, were unseen during the learning phase of the model. Another interesting example concerns RICTOR and EGFR proteins that do not share any known ligand. Starting from the primary sequence of RICTOR, Prot2Drug generated 3 ligands that are known binders of EGFR (CHEMBL1203934, CHEMBL1204360, and CHEMBL542484). It is interesting to investigate the binding affinity between these molecules and RICTOR since the regulation of both EGFR and RICTOR may help in cancer future therapy for glioblastoma.^53^ Noteworthy is the molecule CHEMBL3683258, a known ligand of the Mitogen-activated protein kinase kinase kinase 5 (MAP3K5), which is a serine/threonine kinase that acts as an essential component of the MAP kinase signal transduction pathway. Prot2Drug generated CHEMBL3683258 from a set of 47 primary sequences in the test set therefore unseen in the training phase and this molecule is not known to bind these proteins under the threshold of bioaffinity of 100 nM (SI Appendix Data Set S2). Of interest, the set of genes encoding these 47 proteins was enriched of genes in BioCarta and KEGG Mapk signaling pathways (*P*-value = 4 × 10^–12^, *P*-value = 2 × 10^–8^ respectively on MSigDB gene sets).^54^ The rediscovery of the Mapk signaling pathway indicates that CHEMBL3683258 is a promising candidate ligand of these 47 protein targets that deserves further investigation.

#### Reproduced FDA-Approved Compounds

Starting from specific proteins, Prot2Drug was able to generate molecules that are FDA-approved small molecules with known activity on the input protein. An example of these properly reproduced interactions is the Progesterone Receptor-Testosterone pair. Starting from the protein representation of PGR, Prot2Drug generated the SMILES string of testosterone (CHEMBL386630), a known drug approved that was not present in *D*_100nM_ as a ligand of PGR but whose activity on PGR is reported in the literature. Testosterone was characterized for its agonist activity at human PGR in an in vitro cell-free assay (Chembl ID: CHEMBL5291860)^55^, by time-resolved fluorescence resonance energy transfer method, yielding an affinity at PGR of 92 nM. Another example is the clothiapine (CHEMBL304902) that is an atypical antipsychotic indicated in the treatment of acute and chronic psychosis as well as anxiety that is present in our data set only as a known ligand of D(2) dopamine receptor (DRD2). Prot2Drug reproduced known interactions of clothiapine with other proteins, and these interactions were not present in our data set: Clothiapine was generated by Prot2Drug from the Histamine H1 receptor and D(3) dopamine receptor proteins, and its activity on these targets is reported in the literature. The measured activity values yielded in different experiments and the corresponding references with the Pubmed IDs are reported in ref 56.

Prot2Drug also generated molecules that are FDA-approved small molecules and known binders of proteins different from the ones used for generating them. Since our data suggest these input proteins as candidate targets of these known drugs, they may help identify off-targets, which may result in adverse drug reactions and safety issues or suggest new candidates for drug repurposing. Among these examples of repurposing, we mention the case of the clothiapine that was generated also from the protein representation of NAE1 that binds the β-amyloid precursor protein that is a cell surface protein with signal-transducing properties, and it is thought to play a role in the pathogenesis of Alzheimer’s disease.^57^ The clothiapine-mediated modulation of NAE1, suggested by Prot2Drug, is relevant in the studies focusing on clozapine as a therapeutic drug for the treatment of Alzheimer’s disease patients^58^ and in the analysis of the effects of cloziapine as antipsychotic drug.

Another example of a candidate for repurposing generated by Prot2Drug is agomelatine (CHEMBL10878), a drug with antidepressant properties prescribed for the treatment of depression in adults, that is generated from the protein representation of the human enzyme quinone reductase 2 (NQO2). Diseases associated with NQO2 include breast cancer^59^ and Parkinson’s disease.^60^ Agomelatine has never been studied in relation to NQO2, but this new candidate protein–ligand pair is consistent with previous studies since agomelatine is a melatonin analogue and melatonin is known to target and inhibit NQO2.^61^ In this case, Prot2Drug suggested an interesting molecule targeting NQO2 because it is an approved drug with effects similar to those of melatonin, a known drug against NQO2. Moreover, further evidence supporting the potential modulation of NQO2 by agomelatine is the knowledge of the mechanism of action of the agomelatine on the Melatonin receptor type 1B and type 1A proteins that share a significant number of ligands with NQO2 (13 ligands with *P*-value = 10^–18^ for both). This evidence suggests more investigations into the mechanisms of binding of agomelatine to quinone reductase 2 and its potential inhibition. Moreover, Prot2Drug generated triclocarban (TCC) (CHEMBL1076347), an antimicrobic agent with known adverse effects, starting from the protein representations of the CC chemokine receptors CCR9 and CCR5. TCC is an active drug on the Bifunctional epoxide hydrolase protein (EPHX2) that does not share ligands with CCR9 and CCR5. Recent studies have shown that exposure to TCC, at human exposure-relevant doses, increases the severity of colitis and exacerbates colon tumorigenesis in mice, suggesting that it could be a risk factor of Inflammatory Bowel Disease (IBD) and associated diseases.^62^ Moreover, it is known that CCR9 and CCR5 are involved in the development of IBD and CCR5 and CCR9 antagonists bear therapeutic potential for IBD.^63,64^ Taken together, these results are consistent with the fact that Prot2Drug, generating triclocarban starting from the CCR9 and CCR5 protein representations, suggests a role for TCC in the modulation of these CC chemokine receptors that must be further investigated.

### Molecular Docking

We evaluated the performance of Prot2Drug for each protein by analyzing the quality of the generated molecules. For each protein, we averaged the QED, SA, and ConPLex scores separately and rewarded those proteins generating molecules more likely to be synthesized (lower SA score), with a good drug-likeness (higher QED score), and that showed promising ConPLex scores. Among the top-ranked proteins, two promising drug targets for cancer and neurodegeneration diseases were selected for molecular docking to assess whether the generated molecules could bind the corresponding input proteins. The first selected protein was the nonopioid intracellular receptor Sigma 1 (SigmaR1). SigmaR1 is a chaperone protein at the endoplasmic reticulum (ER) modulating the activity of the stress sensor IRE1 and the cytokine expression.^65^ SigmaR1 has been intensely analyzed over the past several decades due to the therapeutic potential of its ligands in different pathologies, including COVID-19.^66^ Clinical trials investigating its efficacy and safety in the treatment of neurodegenerative diseases including amyotrophic lateral sclerosis and Huntington’s disease are ongoing. Moreover, SigmaR1 ligands are being evaluated for treating and imaging cancer.^67^ Finally, recent studies have shown SigmaR1 to link the SARS-CoV2 replicase/transcriptase complex to ER membranes by binding directly to nonstructural protein 6.^68,69^ There is interest in the rational design of novel SigmaR1 ligands with potent antiviral activity against severe acute respiratory syndrome CoV-2. The role of Sigma1R under different medical conditions makes it a valuable target for drug development. Generated SigmaR1 molecules resulted promising candidates specific for SigmaR1 since ConPLex scores discriminated these SigmaR1 ligands pairs from pairs of SigmaR1 with random generated molecules (AUC = 0.79, 0.77, and 0.59 in TR2, TR1, and TE3, respectively). Moreover, the generated molecules were good candidates due to high drug-likeness (QED = 0.70 ± 0.15, 0.69 ± 0.15, and 0.64 ± 0.19 in TR2, TR1, and TE3, respectively) and low synthetic accessibility score (AS = 2.42 ± 0.6, 2.60 ± 0.7, and 2.92 ± 0.6 in TR2, TR1, and TE3, respectively). We collected all of the new molecules generated by Prot2Drug when SigmaR1 was known to the model and present in TR1 and TR2 as well as when the target was unknown and present in TE1. Analogously, we randomly drawn by TR1, TR2, and TE1 the same number of new molecules generated by Prot2Drug by using different protein targets. We performed docking simulations on these positive and negative protein–ligand pairs (Table 5) by considering only the molecules for which the docking procedure was able to find a docking pose. For each comparison, we tested the hypothesis that the docking scores relative to positive pairs were greater than those relative to negative pairs. To this end, we evaluated the AUC and the *p*-value of the one-sided Mann–Whitney test (see Table 5 and Figure 11A). The results show that the new molecules generated by Prot2Drug have better docking scores than random molecules when the target is known and when it is unknown to the model.

**Figure 11:**
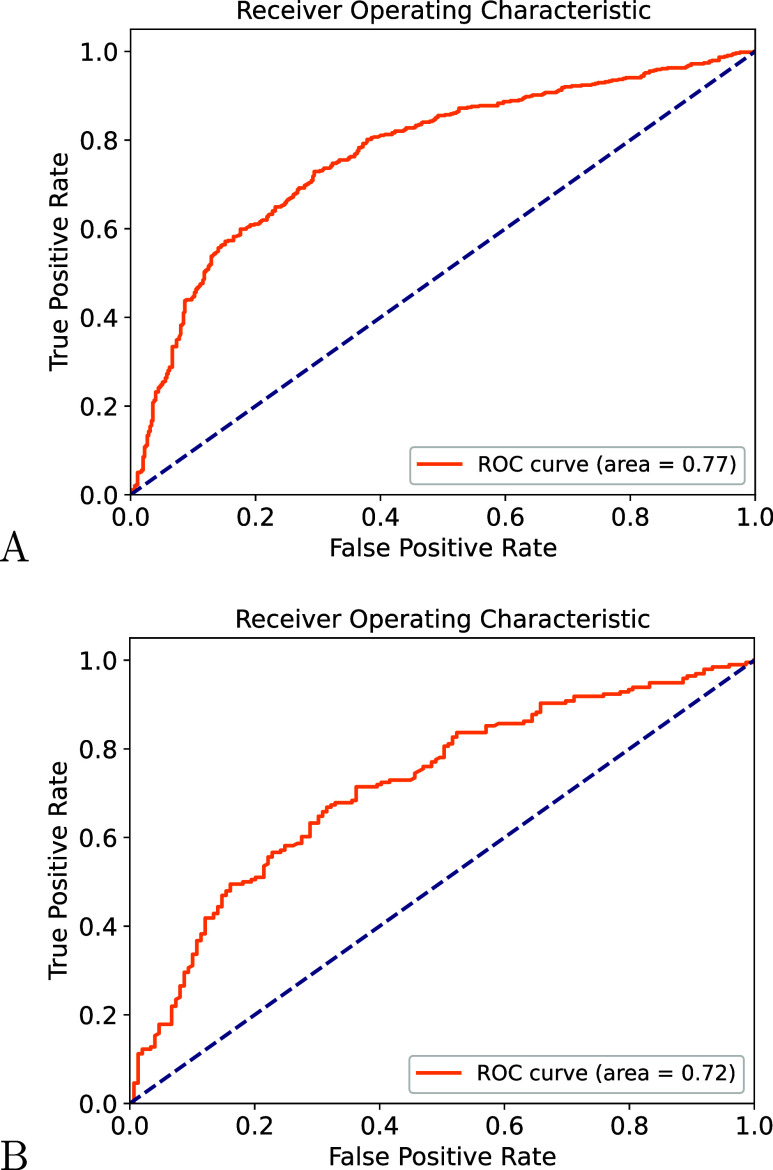
ROC curve illustrating the performance of docking simulations in distinguishing positive and negative protein–ligand pairs. The analysis considers (A) molecules generated by Prot2Drug for SigmaR1 and molecules generated for protein targets different from SigmaR1 and (B) molecules generated by Prot2Drug for PGR and molecules generated for protein targets different from PGR.

**Table 5:** Statistical Assessment of the Docking Scores Computed on the Binding Site of SigmaR1

positive set	negative set	AUC	*p*-value
SigmaR1-TR1	non-SigmaR1-TR1	0.75	1.36 × 10^–49^
SigmaR1-TR2	non-SigmaR1-TR2	0.77	1.80 × 10^–48^
SigmaR1-TE3	non-SigmaR1-TE3	0.61	7.91 × 10^–11^

The second selected protein was the progesterone receptor (PGR) that is involved in breast cancer for its role in both controlling mammary gland tumorigenesis and determining the responsiveness to endocrine therapies.^70^ Given its crucial role, PGR represents a critical therapeutic target for the treatment of breast cancer^71^ and existing progesterone inhibitors may have side effects that limit their use.^70^ For this purpose, developing drugs with greater selectivity for tissues of interest can help reduce side effects. Generated candidate PGR ligands were specific for PGR since ConPLex scores discriminated these PGR-ligand pairs from pairs of PGR with randomly generated molecules (AUC = 0.70, 0.85, and 0.86 in TE1, TR3, and TR2, respectively). Moreover, the generated molecules were comparable with the known ligands of PGR in *D*_100nM_ in terms of drug-likeness (QED = 0.64 ± 0.18, 0.62 ± 0.17, and 0.63 ± 0.17 in TE1, TR2, and TR3, respectively) and synthetic accessibility score (AS = 3.15 ± 0.9, 3.03 ± 0.6, and 3.03 ± 0.7 in TE1, TR2, and TR3, respectively). Also in this case, we collected all of the new molecules generated by Prot2Drug for this protein target in both scenarios when PGR was known to the model and present in TR2 and TR3, and when it was unknown and present in TE1. We performed docking simulations on these positive and negative protein–ligand pairs (Table 6 and Figure 11B) and tested the hypothesis that the docking scores relative to positive pairs were greater than the ones relative to negative pairs. The AUC values and p-values also confirm for this target the potential interaction between PGR and its generated molecules. In the synthesis, these results further highlight the ability of our model to propose novel good drug candidates for a given protein sequence.

**Table 6:** Statistical Assessment of the Docking Scores Computed on the Binding Site of PGR

positive set	negative set	AUC	*p*-value
PGR-TE1	non-PGR-TE1	0.66	5.16 × 10^–08^
PGR-TR2	non-PGR-TR2	0.72	1.73 × 10^–12^
PGR-TR3	non-PGR-TR3	0.69	4.83 × 10^–10^

## Discussion

In this work, we introduced Prot2Drug a novel generative deep learning model which generates ligands binding a given protein target by using a distributed representation of the protein. The model is a simple two-layer transformer decoder that maps the representation provided by a pretrained protein language model (PLM) to molecules binding the input protein target. The proposed approach fits into the general framework suggested in ref 14 but with a substantial difference. Instead of using the protein’s amino acid sequence as input, we used its distributed representation learned by a PLM trained on millions of protein sequences. Prot2Drug was trained by using thousands of known protein–ligand interactions provided by the ChEMBL repository. The excellent results provided by Prot2Drug indicate that the protein distributed representation carries relevant information concerning the structure and binding pockets of protein targets. Notice that the used protein embedding was learned independently of the problem at hand. In other words, no information concerning the ligands of the protein was used. The embedding unveils the receipt to follow for designing molecules binding a given protein target, and the transformer translates such instructions by using the syntax of the molecular language, generating compounds binding the target with superior physicochemical properties. Indeed, the new molecules exhibit QED and SA values even better than the ones used for training, as our statistical analysis indicates. The values of internal diversity and maximum Tanimoto similarity indicate that Prot2Drug spans unexplored regions of the chemical space. The properties of the generated molecules are not an artifact of our results. They indicate the expressive power of the adopted PLM and the ability of the transformer to capitalize on the knowledge gathered from thousands of protein–ligand interactions. The surprising number of reproduced compounds deserves an in-depth analysis. Prot2Drug generated numerous molecules with binding affinities lower than 100 nM not included in the training sets, by using never seen before proteins. Prot2Drug reproduced not only known proper ligands, compounds binding the protein used for generating them, but also ligands suggesting new repurposing strategies. The existence in our results of repurposed ligands as well as ligands generated by multiple different protein targets is a consequence of the fact that the ligand-protein mapping follows a power law^72^ indicating that few ligands bind tens of protein domains, whereas most ligands bind only one. We conjecture that proteins generating the same ligands have binding pockets with similar structures.

As mentioned in the Results section, Prot2Drug was capable of generating molecules that have been proven to be bioactive based on past experiments. Remarkably, the reproduction of proper ligands is particularly indicative of Prot2Drug’s ability to generate good candidates for targets for which neither the 3D structure nor experimental information related to other ligands was available. In SI Appendix Figure S14, we show the 2D structures along with the corresponding targets and experimental affinity data for a representative pool of molecules reproduced by Prot2Drug. It is notable how our model can generate molecules that belong to different regions of chemical space and are affine to targets belonging to different pharmacological classes. The analysis of potential candidates for repurposing was particularly noteworthy. In many cases (∼50%), the generated molecules exhibited a high degree of novelty compared to molecules known to be active against the input target (i.e., Tanimoto similarity calculated using circular Morgan fingerprints with a radius of 2 was less than 0.6). In particular, Prot2Drug generated more different molecules for never seen before protein targets with average maximum similarity scores of 0.27, 0.28, and 0.29 for TE1, TE2, and TE3, respectively. In other words, these molecules are not merely simple analogues of known active molecules for the input target, making the suggestion of potential repurposing unlikely to be derived from human observation of the chemical structures alone. For instance, using the primary sequence of Glycogen synthase kinase-3 β, a target whose dysfunction is associated with bipolar disorder, Prot2Drug generated a molecule (CHEMBL129199) known to be active on the Histamine H4 receptor (*K_i_* = 38.0 nM^73^), a target involved in allergies, asthma, and autoimmune diseases. The compound, whose 2D structure is shown in SI Figure S15, is structurally different from the molecules known to bind Glycogen synthase kinase-3 β, with the computed similarity (Tc) to the most similar active compound equal to 0.33. Another example is provided by CHEMBL64192, a compound generated by Prot2Drug from the primary sequence of the Ephrin type-B receptor 4 (2D structure depicted in SI Appendix Figure S15), a protein involved in vascular development. This compound has a chemical structure different from molecules commonly known to be active on this target (Tanimoto coefficient calculated against the most similar molecule equal to 0.35) and is known to be active on another target, Mitogen-activated protein kinase 14 (56.0 nM),^74^ a protein that plays an essential role in the cardiovascular system as a regulator of cytokines in myocytes. It is worth noting that this type of analysis not only indicates the potential repurposing of the generated molecules but also suggests a similarity between the two targets, making it reasonable to attempt multiple repurposing. This means taking all active molecules from one target and testing them against the other. Furthermore, when identifying targets with synergistic pharmacological activities, Prot2Drug could suggest new associations for designing novel multitarget compounds.

The generative method presented in this work shows the exceptional expressive power of PLM able to extract relevant information concerning the ligand binding pockets of the protein targets starting from the primary sequence only. The integration of ProtTrans embeddings with transformer models enables us to accurately capture the lexicon of proteins that bind specific small molecules. Remarkably, Prot2Drug enables the design of promising ligands even for protein targets with limited or no information about their ligands or 3D structure.

## Supplementary Material















## Data Availability

The known protein–ligand interactions were acquired from the public database ChEMBL (https://chembl.gitbook.io/chembl-interface-documentation/downloads). Our data and code are available at https://doi.org/10.5281/zenodo.14637195.

## Notes

Detailed analysis of the physicochemical properties of the new molecules, the predicted binding affinity between generated compounds and target proteins, and reproducibility of known ligands (PDF)
List of known proper interactions reproduced by Prot2Drug by using distributed representations of proteins in test set 1, test set 2, and test set 3 (XLSX)
List of known repurposed interactions reproduced by Prot2Drug by using distributed representations of proteins in test set 1, test set 2, and test set 3 (XLSX)
List of known repurposed interactions reproduced by Prot2Drug by using distributed representations of proteins in training set 1 (XLSX)
List of known repurposed interactions reproduced by Prot2Drug by using distributed representations of proteins in training set 2 (XLSX)
List of known repurposed interactions reproduced by Prot2Drug by using distributed representations of proteins in training set 3 (XLSX)
List of new molecules generated by Prot2Drug by using protein targets in TR1, TR2, TR3, TE1, TE2, and TE3 with a ConPLex score *s* ≥ 0.923 together with QED and SA values (XLSX)
