# Supplementary material for: Transformer Decoder Learns from a Pretrained Protein Language Model to Generate Ligands with High Affinity
Source: J Chem Inf Model. 2025 Jan 28;65(3):1258–77. doi: 10.1021/acs.jcim.4c02019 (PMC12524004; doi:10.1021/acs.jcim.4c02019)
Supplement: Supplementary file 1 [file ci4c02019_si_001.pdf]

Supporting Information

Transformer decoder learns from a pretrained protein language model to generate ligands with high affinity

Teresa Maria Creanza[1], Domenico Alberga[2], Cosimo Patruno[1], Giuseppe Felice Mangiatordi[2], Nicola Ancona[1*]

[1*]Institute of Intelligent Industrial Technologies and Systems for Advanced Manufacturing, Consiglio Nazionale delle Ricerche, Via G. Amendola, 122/d, Bari, 70126, Italy.
[2]Institute of Crystallography, Consiglio Nazionale delle Ricerche, Via G. Amendola, 122/d, Bari, 70126, Italy.

*Corresponding author(s). E-mail(s): nicola.ancona@cnr.it;

1 Sequence similarities of protein targets

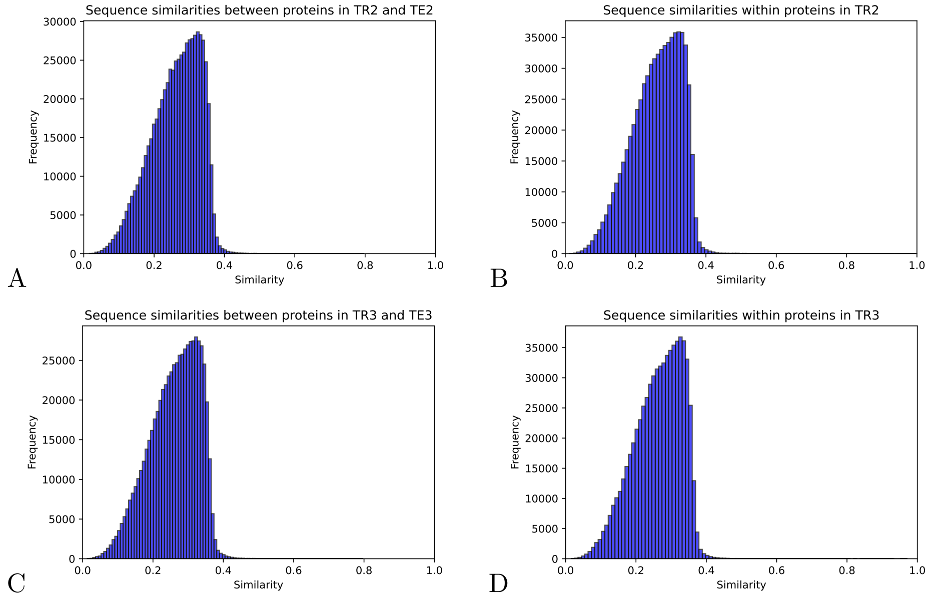

Fig. S1 Distribution of sequence similarities between protein pairs. A) Sequence similarities between proteins belonging to TR2 and TE2; B) sequence similarities of proteins within TR2; C) sequence similarities between proteins belonging to TR3 and TE3; D) sequence similarities of proteins within TR3.

The distribution of pairwise sequence similarities between proteins belonging to TR2 and TE2, as well as to TR3 and TE3 are depicted in Fig.S1A,C respectively. Notably, the majority of the sequences in the training set have a similarity less than 40% with the ones in the test set. The same consideration holds true for protein pairs within TR2 and TR3 (see Fig.S1B,D). To assess the similarities between proteins we used the Needleman-Wunsch global alignment algorithm of the BioPython package.

2 Physicochemical properties of new molecules

We show the plots concerning the physicochemical properties of the new molecules generated by Prot2Drug by using known protein targets belonging to training sets TR1, TR2, TR3, as well as never seen before proteins belonging to test sets TE1, TE2 and TE3. The comparisons between the new molecules and the ones belonging to D100nM were statistically assessed. In particular, the comparisons concerning the quantitative estimation of drug-likeness (QED), the synthetic accessibility (SA) the internal diversity, the LogP and the molecular weight were assessed by using one-sided Mann-Whitney tests (Fig.S2, Fig.S3, Fig.S4, Fig.S6, Fig.S7). The histograms of the average maximum Tanimoto similarity of the generated molecules are depicted in

Fig.S5. Moreover, the correlation between average QED and SA scores measured on the new ligands and the ones in D100nM was assessed by using the Pearson Correlation Coefficient (PCC) (Fig.S8, Fig.S9).

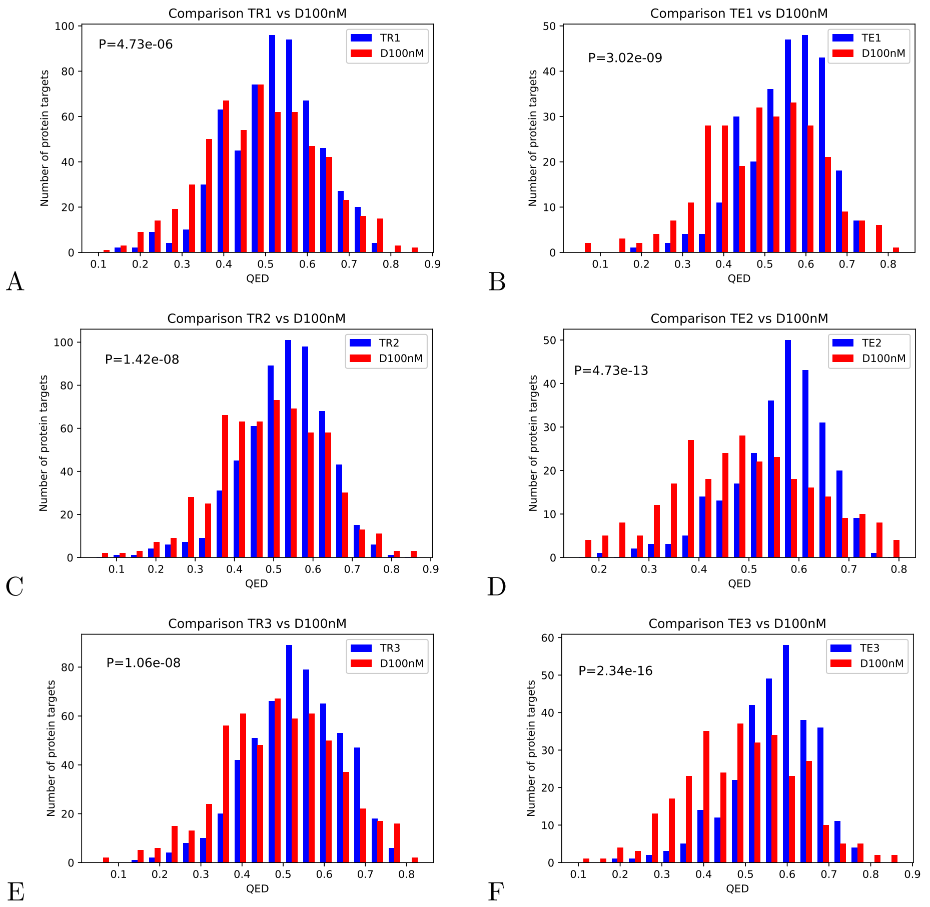

Fig. S2 Histograms of the quantitative estimation of drug-likeness (QED) scores measured on the new molecules generated by using the ProtTrans distributed representation of the protein targets belonging to training and test sets (blue) and on the molecules in D100nM (red).

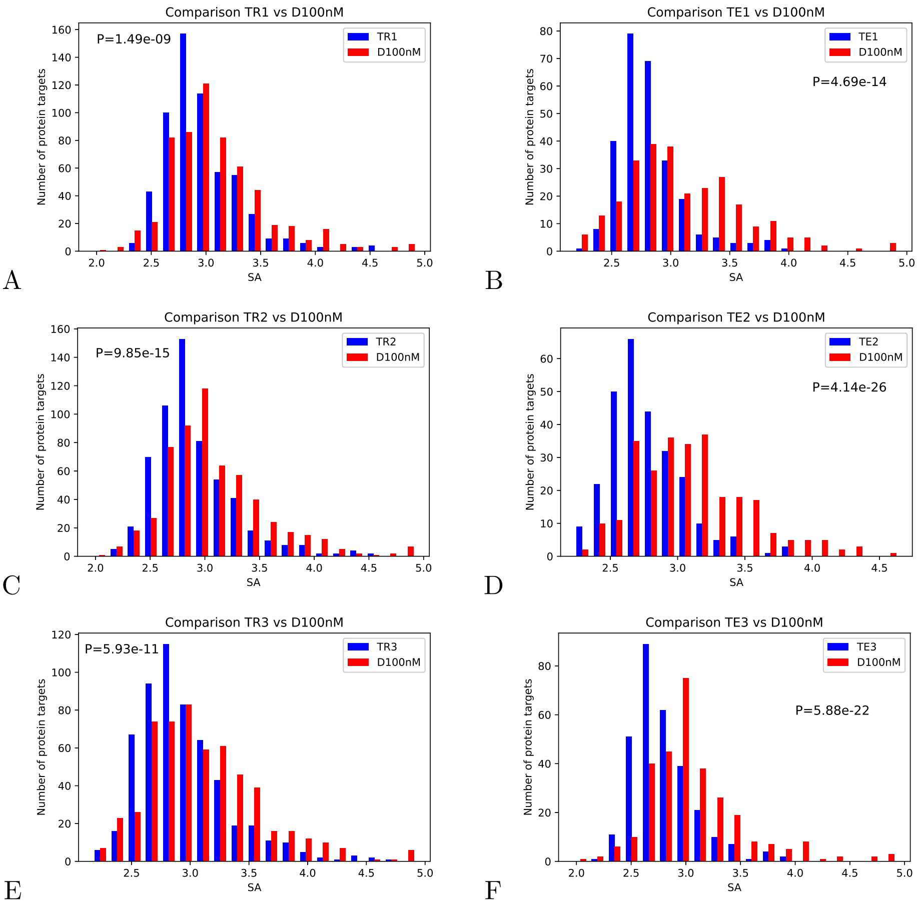

Fig. S3 Histograms of the synthetic accessibility (SA) scores measured on the new molecules generated by using the ProtTrans distributed representation of the protein targets belonging to training and test sets (blue) and on the molecules in D100nM (red).

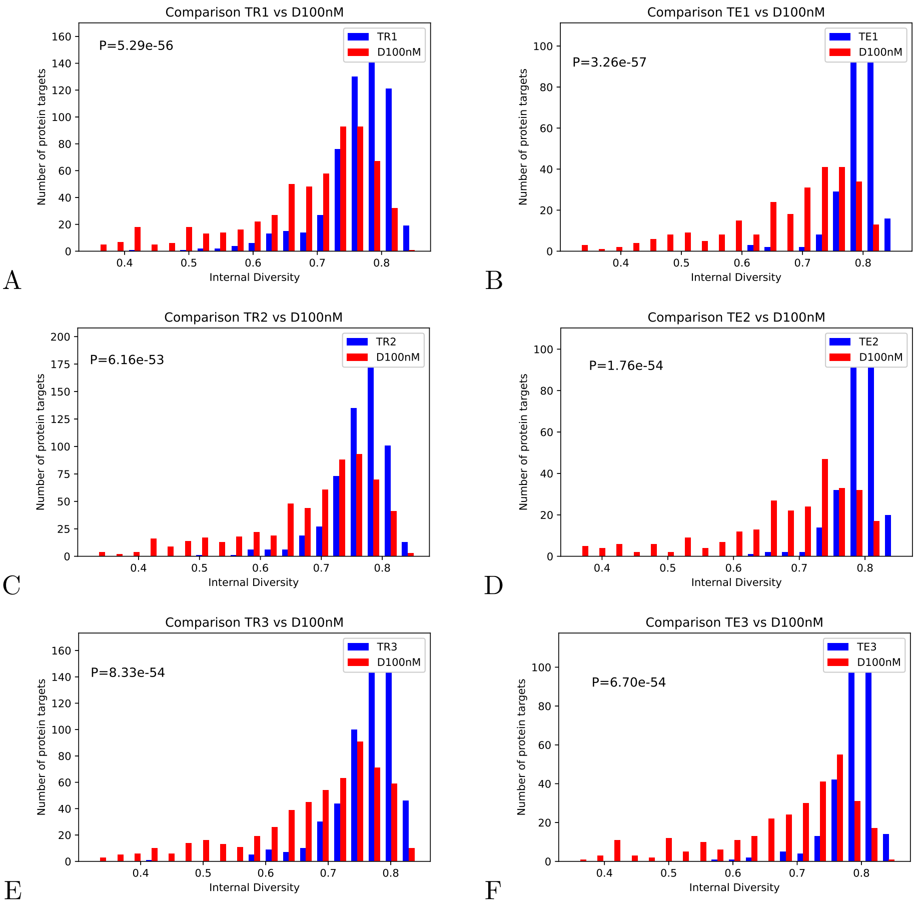

Fig. S4 Histograms of the internal diversity scores measured on the new molecules generated by using the ProtTrans distributed representation of the protein targets belonging to training and test sets (blue) and on the molecules in D100nM (red).

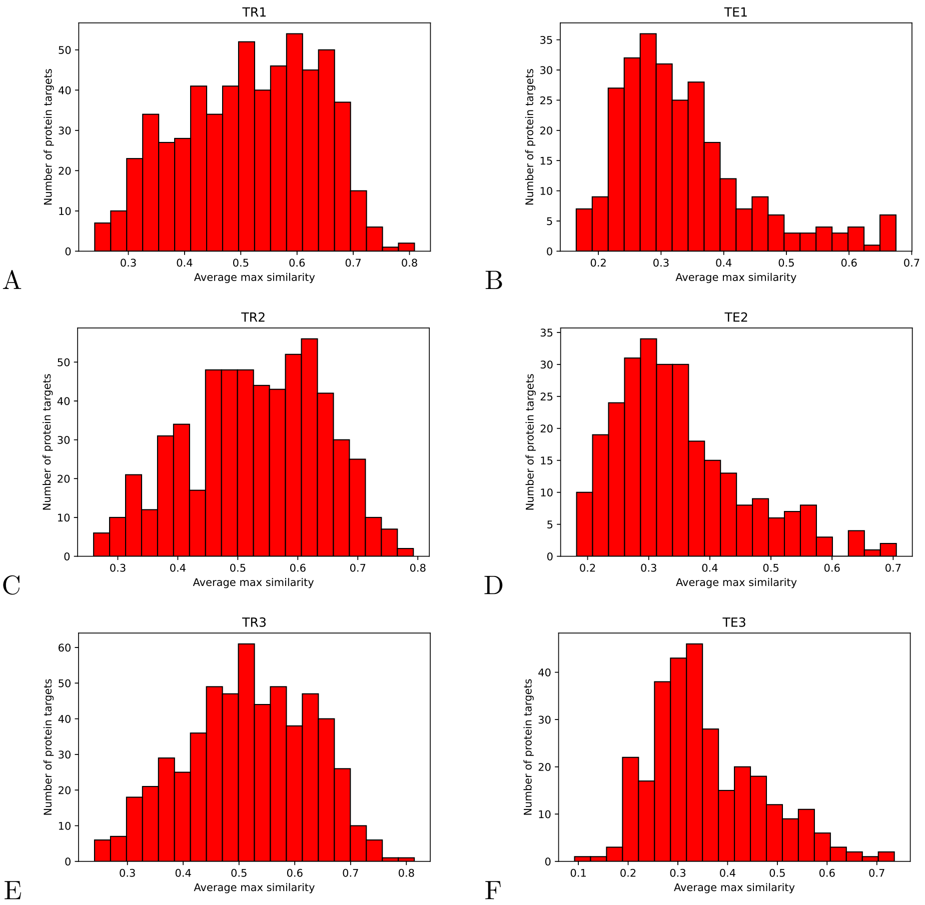

Fig. S5 Histograms of the average Maximum Tanimoto similarity scores considering for each generated molecule the most similar molecule in the reference set active on the target protein sequence.

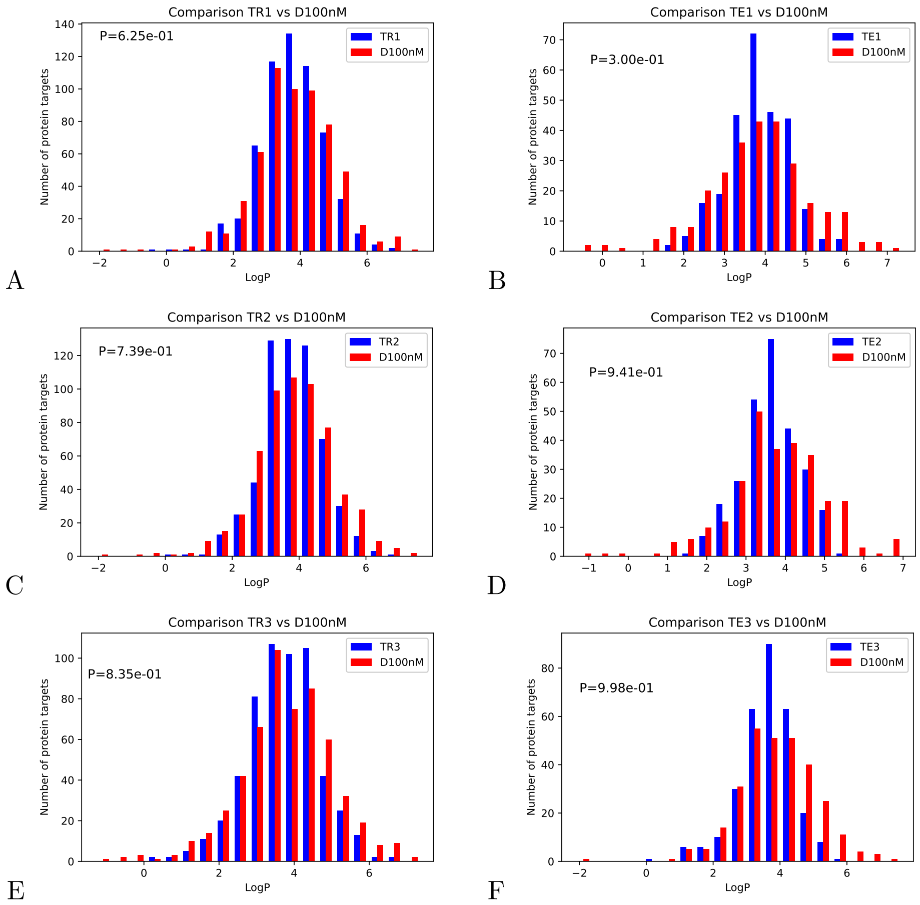

Fig. S6 Histograms of the average logP values measured on the new molecules generated by using the ProtTrans distributed representation of the protein targets belonging to training and test sets (blue) and on the molecules in D100nM (red).

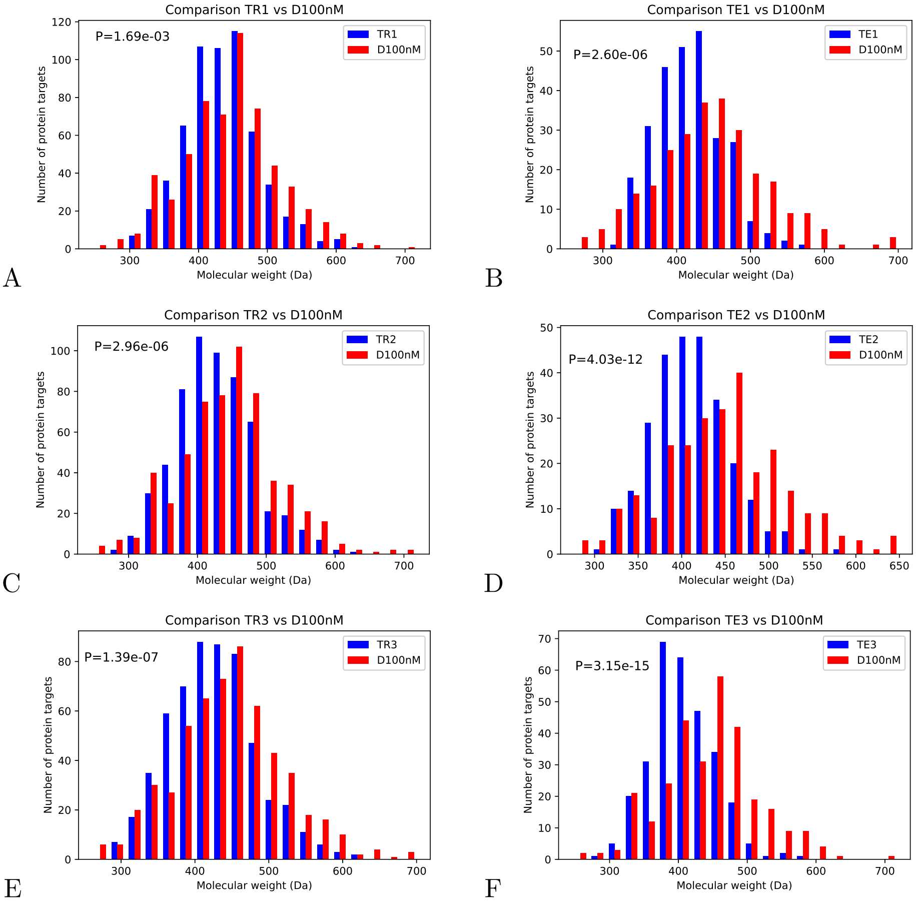

Fig. S7 Histograms of the average molecular weight values measured on the new molecules generated by using the ProtTrans distributed representation of the protein targets belonging to training and test sets (blue) and on the molecules in D100nM (red).

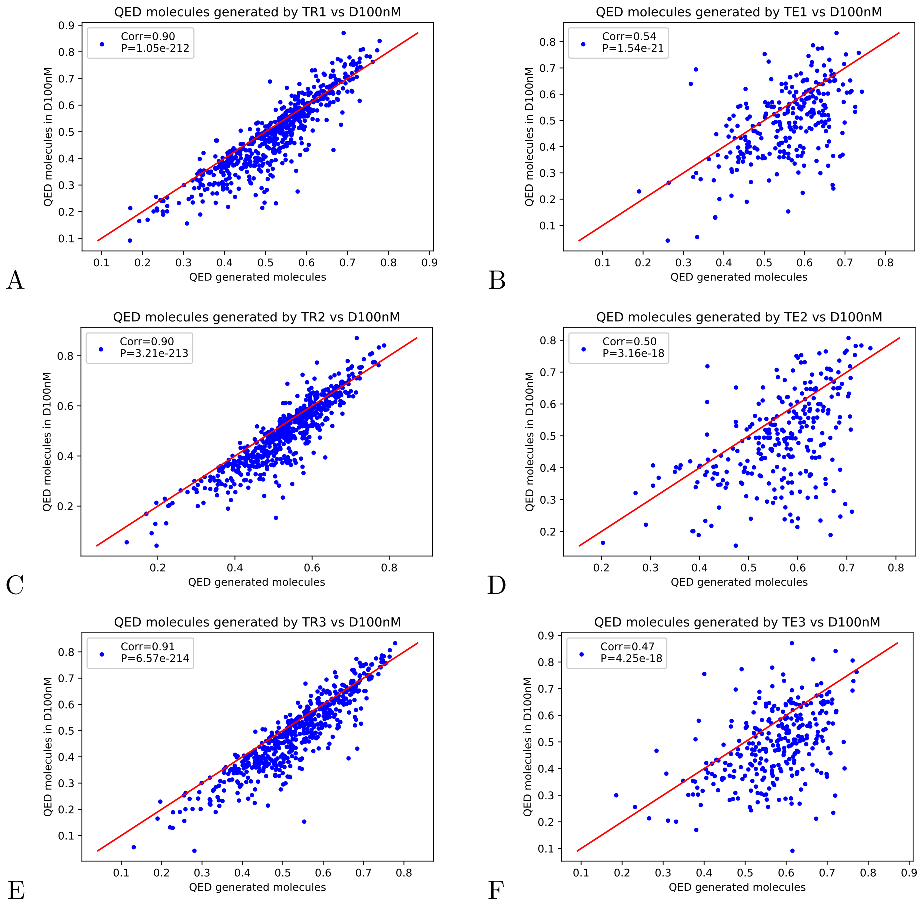

Fig. S8 Scatter plots of the average QED scores for each protein. The values were measured on the new molecules generated by using the ProtTrans distributed representation of the protein targets belonging to training and test sets.

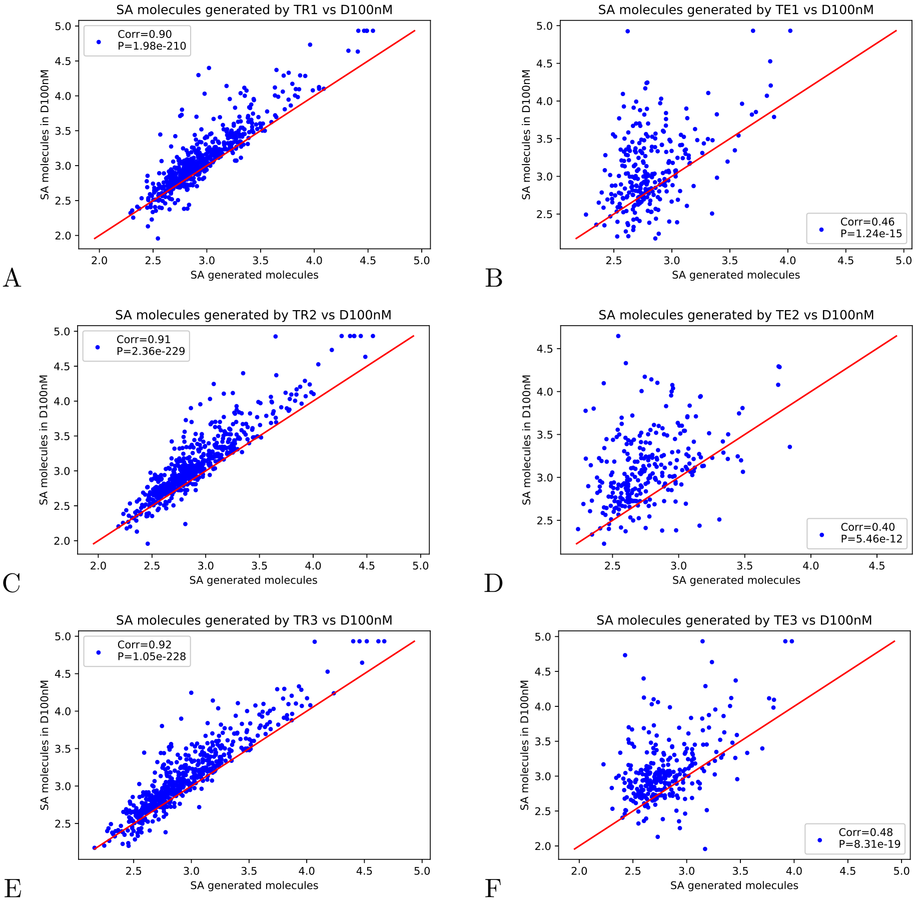

Fig. S9 Scatter plots of the average SA scores for each protein. The values were measured on the new molecules generated by using the ProtTrans distributed representation of the protein targets belonging to training and test sets.

3 Testing the predicted binding affinity between generated compounds and target proteins

3.1 New molecules generated by M_2 by using proteins in the training set TR2

Comparison a. Comparing the sets POS_G versus NEG_G, we found 607 on 1156 protein targets with P≤2.58E-02 and false discovery rate FDR=5% (see Fig.S10A). Moreover, comparing these AUC values with the ones evaluated on D_{100nM} for the same protein targets (see Methods section), we measured a correlation of PCC=0.83 (P=3E-183) (see Fig.S10B).
Comparison b. Comparing the sets POS_G versus NEG_{D100nM}, we found 645 proteins with P≤2.77E-02 at FDR=5% (see Fig.S10C). Also in this case, the AUC values correlated with the ones evaluated on D_{100nM} for the same protein targets (PCC=0.82 with P=9E-175, Fig.S10D).
Comparison c. Comparing the sets POS_G versus TRUE_POS, and considering the set of protein targets having a number of known ligands $\geq$ 10 in D_{100nM}, we found a mean value of AUC and standard deviation of 0.48±0.10 indicating that new molecules and known ligands had comparable predicted binding scores assessed on the same protein targets (see Fig.S10E).

3.2 New molecules generated by M_2 by using proteins in the test set TE2

Comparison d. Comparing the sets POS_G versus NEG_G, we found 305 on 558 protein targets with statistically significant AUC values with P≤2.57E-02 at FDR=5% (see Fig.S11A). Also in this comparison, the AUC values were correlated with the ones evaluated on D_{100nM} for the same protein targets, although with a lower correlation value (PCC=0.58 with P=5E-32, Fig.S11B).
Comparison e. Comparing the sets POS_G versus NEG_{D100nM}, we found 338 protein targets with P≤2.45E-02 at FDR=5% (see Fig.S11C). The AUC values were correlated with the ones evaluated on D_{100nM} for the same protein targets (PCC=0.57 with P=9E-30, Fig.S11D).
Comparison f. Comparing the sets POS_G versus TRUE_POS, and considering the set of protein targets having a number of known ligands $\geq$ 10 in D_{100nM}, we found a mean value of AUC and standard deviation of 0.47±0.15 confirming that new molecules and known ligands had comparable predicted binding scores assessed on the same protein targets (see Fig.S11E).

3.3 New molecules generated by M_3 by using proteins in the training set TR3

Comparison a. Comparing the sets POS_G versus NEG_G, we found 589 on 1156 protein targets with P≤2.52E-02 and false discovery rate FDR=5% (see Fig.S12A). Moreover, comparing these AUC values with the ones evaluated on D_{100nM} for the

same protein targets (see Methods section), we measured a correlation of PCC=0.84 (P=4E-185) (see Fig.S12B).

Comparison b. Comparing the sets POS_G versus NEG_{D100nM}, we found 634 proteins with P≤2.62E-02 at FDR=5% (see Fig.S12C). Also in this case, the AUC values correlated with the ones evaluated on D_{100nM} for the same protein targets (PCC=0.83 with P=9E-179, Fig.S12D).

Comparison c. Comparing the sets POS_G versus TRUE_POS, and considering the set of protein targets having a number of known ligands ≥ 10 in D_{100nM}, we found a mean value of AUC and standard deviation of 0.48±0.10 indicating that new molecules and known ligands had comparable predicted binding scores assessed on the same protein targets (see Fig.S12E).

3.4 New molecules generated by M_3 by using proteins in the test set TE3

Comparison d. Comparing the sets POS_G versus NEG_G, we found 297 on 553 protein targets with statistically significant AUC values with P≤2.67E-02 at FDR=5% (see Fig.S13A). Also in this comparison, the AUC values were correlated with the ones evaluated on D_{100nM} for the same protein targets, although with a lower correlation value (PCC=0.58 with P=5E-32, Fig.S13B).

Comparison e. Comparing the sets POS_G versus NEG_{D100nM}, we found 303 protein targets with P≤2.68E-02 at FDR=5% (see Fig.S13C). The AUC values were correlated with the ones evaluated on D_{100nM} for the same protein targets (PCC=0.56 with P=2E-30, Fig.S13D).

Comparison f. Comparing the sets POS_G versus TRUE_POS, and considering the set of protein targets having a number of known ligands ≥ 10 in D_{100nM}, we found a mean value of AUC and standard deviation of 0.47±0.15 confirming that new molecules and known ligands had comparable binding scores assessed on the same protein targets (see Fig.S13E).

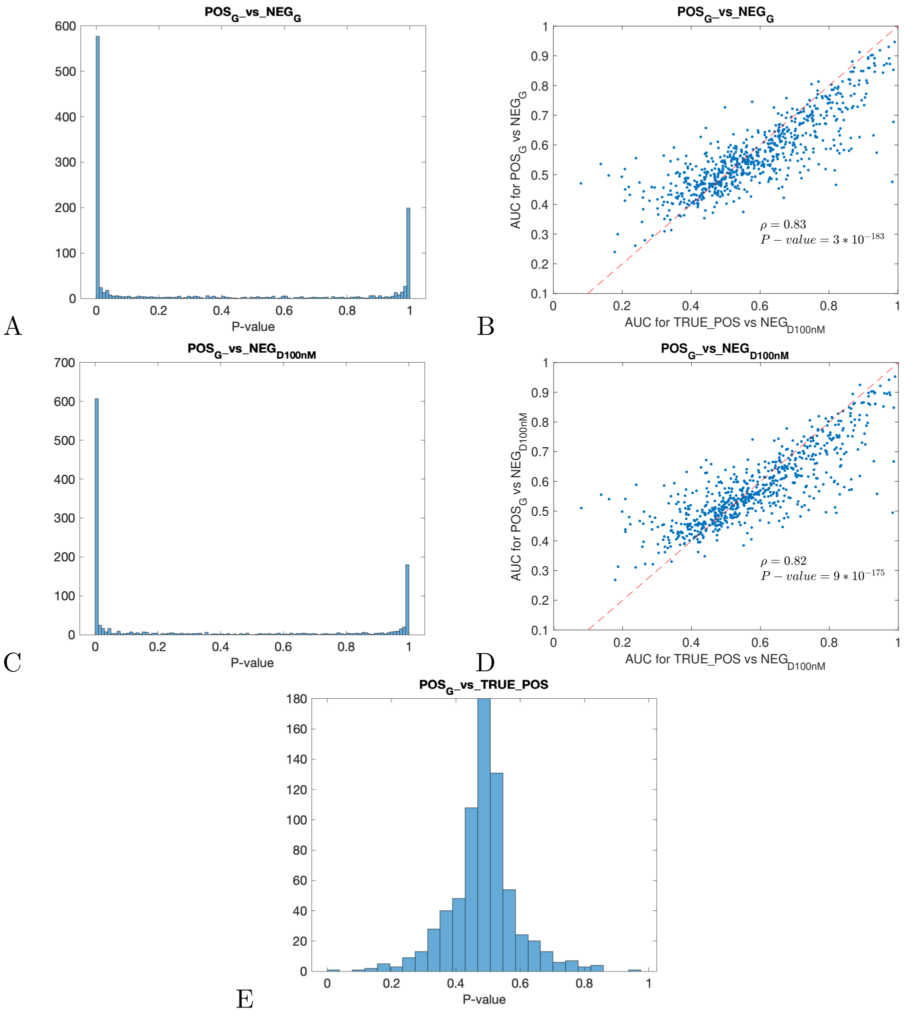

Fig. S10 Statistical assessment of the AUC values evaluated by using the new molecules generated by using the protein targets in the training set TR2. A) and C): P-value histograms of the Mann-Whitney test in the Comparisons a and b respectively. B) and D): scatter plots of the AUC values: x-axis represents the values measured by using the known protein-ligands interactions in D100nM for each protein and y-axis represents the values measured in the Comparisons a and b respectively. E) Histogram of the AUC values evaluated in the Comparison c.

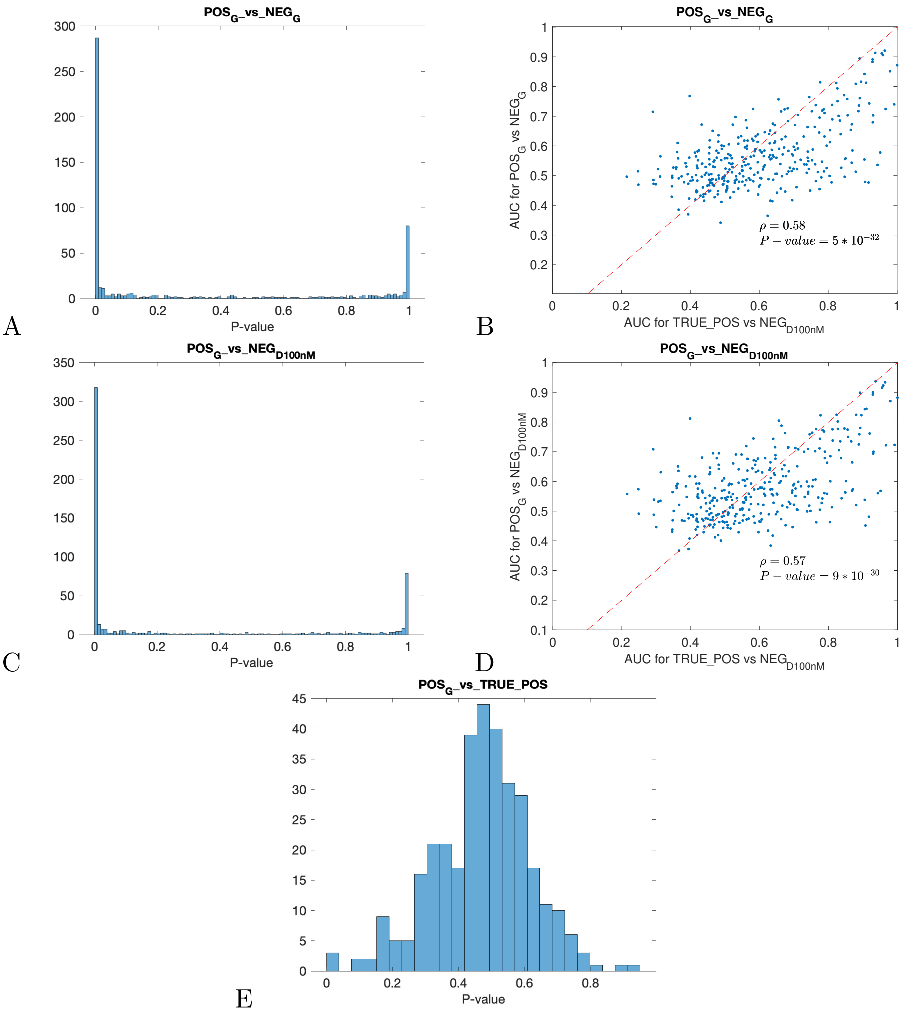

Fig. S11 Statistical assessment of the AUC values evaluated by using the new molecules generated by using the protein targets in the test set TE2. A) and C): P-value histograms of the Mann-Whitney test in the Comparisons d and e respectively. B) and D): scatter plots of the AUC values: x-axis represents the values measured by using the known protein-ligands interactions in D100nM for each protein and y-axis represents the values measured in the Comparisons d and e respectively. E) Histogram of the AUC values evaluated in the Comparison f.

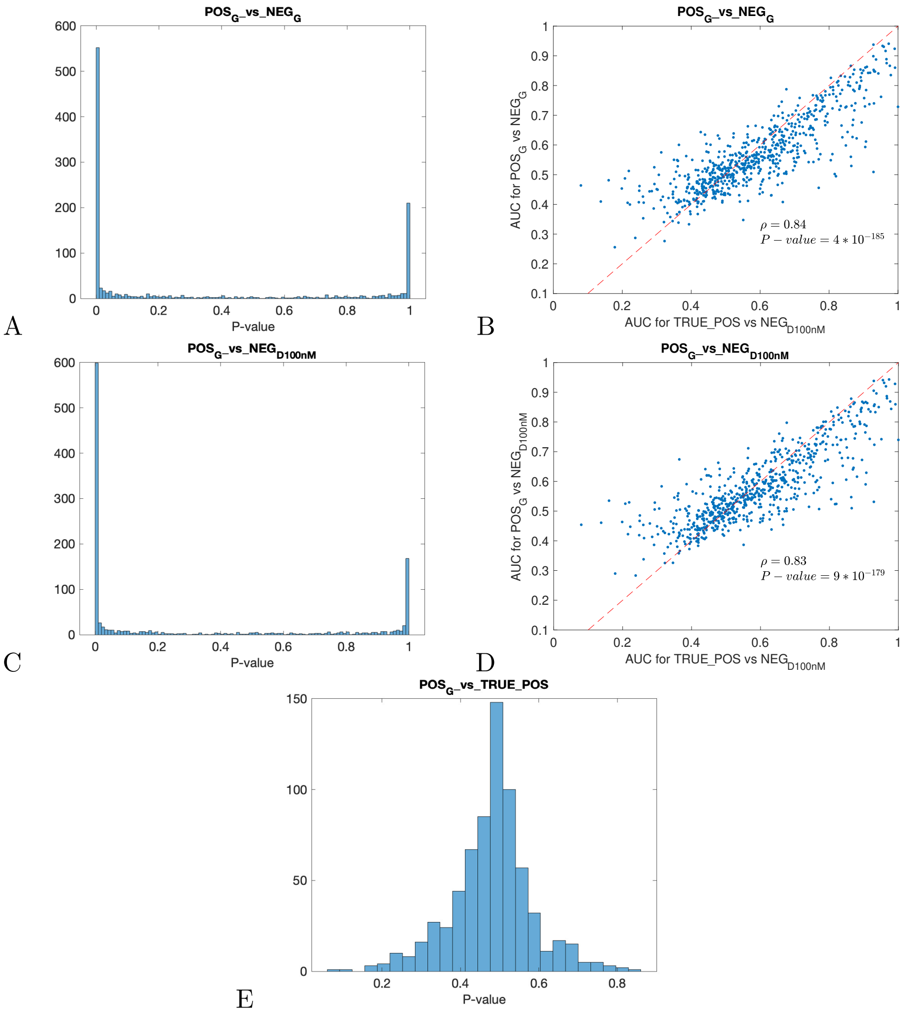

Fig. S12 Statistical assessment of the AUC values evaluated by using the new molecules generated by using the protein targets in the training set TR3. A) and C): P-value histograms of the Mann-Whitney test in the Comparisons a and b respectively. B) and D): scatter plots of the AUC values: x-axis represents the values measured by using the known protein-ligands interactions in D100nM for each protein and y-axis represents the values measured in the Comparisons a and b respectively. E) Histogram of the AUC values evaluated in the Comparison c.

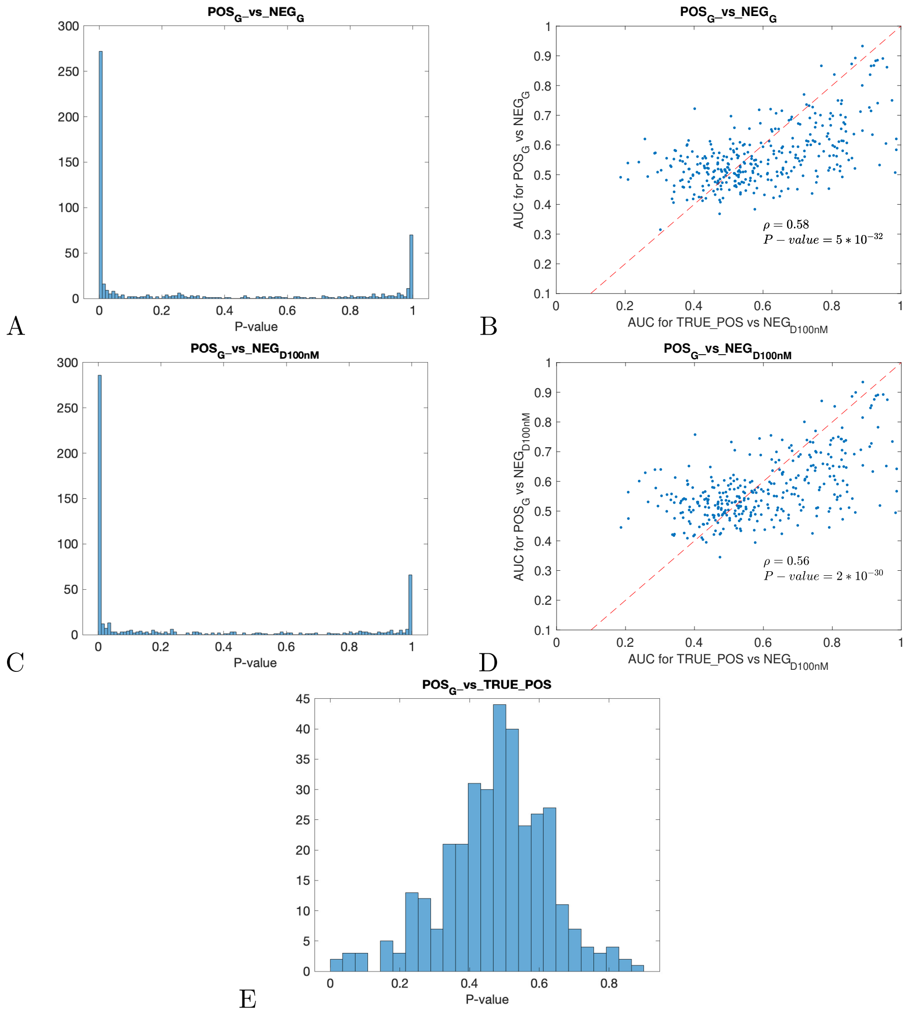

Fig. S13 Statistical assessment of the AUC values evaluated by using the new molecules generated by using the protein targets in the test set TE3. A) and C): P-value histograms of the Mann-Whitney test in the Comparisons d and e respectively. B) and D): scatter plots of the AUC values: x-axis represents the values measured by using the known protein-ligands interactions in D100nM for each protein and y-axis represents the values measured in the Comparisons d and e respectively. E) Histogram of the AUC values evaluated in the Comparison f.

4 Reproducibility of known ligands

Examples of 2D structures with the ChEMBLID and affinity data of a representative pool of molecules reproduced by Prot2Drug are shown in Fig.S14 and Fig.S15.

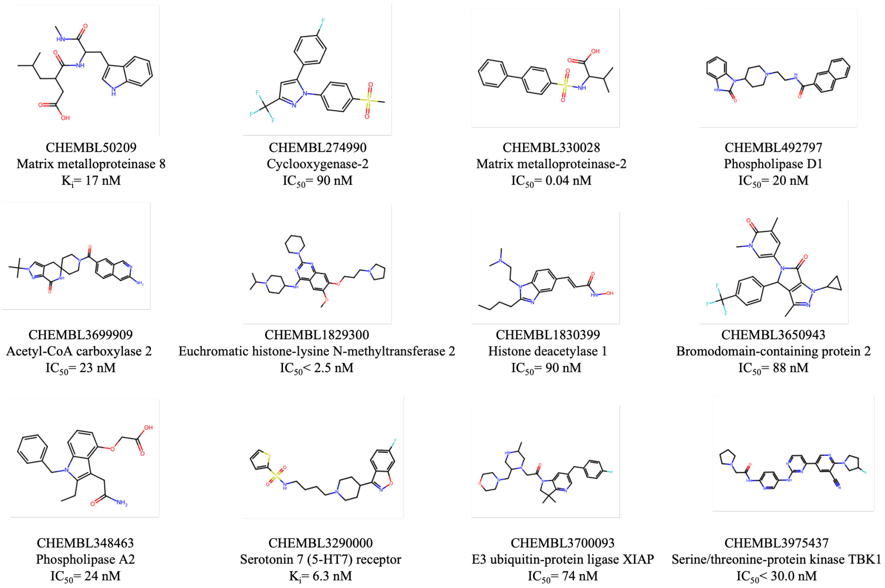

Fig. S14 2D structures, CHEMBLID and affinity data of a representative pool of molecules reproduced by Prot2Drug starting from a given protein target belonging to the test set and known to possess high affinity towards the same target.

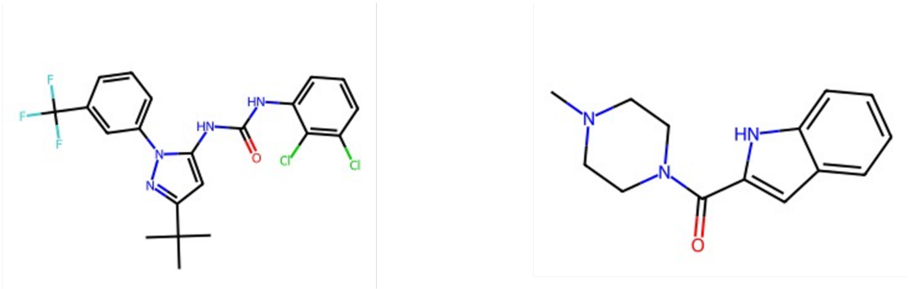

CHEMBL64192 CHEMBL129199

Fig. S15 2D structures and CHEMBLID of two representative molecules generated by Prot2Drug from a given protein sequence (Ephrin type-B receptor 4 and Glycogen synthase kinase-3 beta for CHEMBL64192 and CHEMBL129199, respectively) but found to be highly affine (IC50 or Ki < 100 nM) toward other targets (Mitogen-activated protein kinase 14 and Histamine H4 receptor, respectively).
